# Federated Time‐to‐Event Data Analysis Using Piecewise Exponential Regression in DataSHIELD

**DOI:** 10.1002/sim.70789

**Published:** 2026-10-18

**Authors:** Bodil Svennblad, Benedicte Delcoigne, Paul Burton, Demetris Avraam

**Affiliations:** ^1^ Department of Surgical Sciences, Medical Epidemiology Uppsala University Uppsala Sweden; ^2^ Department of Medicine, Clinical Epidemiology Division Karolinska Institute Stockholm Sweden; ^3^ Population Health Sciences Institute Newcastle University Newcastle upon Tyne UK; ^4^ Section of Epidemiology, Department of Public Health University of Copenhagen Copenhagen Denmark

**Keywords:** DataSHIELD, federated time‐to‐event, piecewise exponential

## Abstract

Time‐to‐event analyses on individual patient data are commonly performed with Cox or Poisson regression. The statistical power of epidemiological findings from such analyses is often sufficient when a large amount of data is analyzed, especially when studying rare outcomes or rare exposures. The increase of sample size can be achieved by co‐analyzing data collected from different cohorts and hosted at different locations. For co‐analyzing data located at different centers, the common options are either (i) to physically transfer and pool the data into a single dataset and analyze them jointly or (ii) to conduct a two‐step meta‐analysis where a separate analysis is done at each center and the center‐specific results are then pooled and combined into a single estimate. DataSHIELD offers a third option where individual patient data from different locations are analyzed in a federated manner without transporting any data. In this paper, we describe how a federated Poisson regression can be conducted on time‐to‐event data using this infrastructure, providing relevant estimates.

## 1 Introduction

To achieve significant advances and accelerate improvements in health‐related topics, the use of resources for scientific research purposes should be maximized [1]. When it concerns epidemiological study data, and moreover in the case of rare outcomes or rare exposures, large or even very large sample sizes are needed to provide adequate statistical power for answering the research questions. When data from a single center do not suffice, it can be helpful or even necessary to use and co‐analyze data from multiple centers. This can be done either by harmonizing and pooling data from multiple locations in a single dataset and then conducting a joint analysis on the pooled data, or by gathering and meta‐analyzing the estimates obtained from multiple studies performed at different locations with similar data (but from different individual subjects) analyzed using the same statistical methods [2]. Because individual‐level data commonly include sensitive information, the first suggested solution of physical data pooling usually raises important ethical and legal issues, which may prohibit sharing such data [3]. The meta‐analysis solution does not face these issues but optimally requires the statistical analyses to be specified in advance which cannot easily be done for complex investigations. The meta‐analysis approach is therefore dependent on the summary statistics already available from the gathered studies. Moreover, statistical power could also be an issue in some studies if the number of individuals with the outcome is too low to provide reliable statistical estimates. A third solution, which overcomes these limitations, exists and is called federated analysis [4]. This approach allows analysis of data from multiple studies simultaneously, without the need for sharing or pooling the individual‐level data, while still providing the same statistical estimates as if the data were physically pooled and analyzed jointly.

Different methods and tools for federated analysis have been developed over the last two decades [5, 6, 7, 8, 9]. These methods are based on the fact that most statistical estimates can be calculated using summary statistics, which by definition do not include individual‐level information, hence minimizing the risk for personal data disclosure. DataSHIELD is one of the tools developed for performing such federated analyses [3, 6, 10, 11, 12]. By using a distributed computing architecture for parallelized analysis, it enables full joint analysis of individual‐level data from several centers, which never leave and are never visible outside the firewall of their local server. Moreover, DataSHIELD does not require major informatics infrastructure and, as it is based on non‐commercial open‐source software, it is very cost‐effective and locally installable. Functions for generalized linear models (GLM) have been implemented in DataSHIELD and Jones et al. demonstrated why and how the regression results obtained from the GLM procedure applied within DataSHIELD on data located at different locations (data centers) are fully equivalent to the results that would be obtained if the datasets were physically pooled and jointly analyzed [3]. While GLM procedures are a mainstay of statistical methods for analyzing epidemiological data, they do not include Cox regression for analyzing time‐to‐event—also called survival—data, which are widely used in (pharmaco‐) epidemiological studies.

A meta‐analysis approach fitting separate Cox models in each center is available within DataSHIELD through the *dsSurvival* package [13, 14]. Algorithms also exist for the Cox regression of federated individual‐level data analyzed as if it was pooled for example, WebDISCO [15], ODAC [16], ODACH [17], WICOX [18] and DC‐COX [19]. They are all privacy‐preserving in the sense that only aggregated data are shared to a central center but all except ODACH also share the individual event times, which can be viewed as potentially disclosive. ODAC and ODACH estimate surrogate log‐likelihood functions and are thereby efficient, as they do not need iterative communication with the centers but they do require a designated lead center with full access to the individual‐level data at that center.

The piecewise exponential model (PEM) assumes constant hazard within disjunct intervals of the follow‐up. The likelihood function coincides with the one from a certain Poisson model which can be used to estimate the coefficients of the PEM [20, 21]. The baseline hazard function is continuous (and left out of the estimation) in a Cox model but a step function in the PEM. An almost non‐parametric model can be estimated by increasing the number of intervals, and the estimates from the PEM approximate those from the Cox model [21]. For federated individual‐level data analysis, there is a gap between the non‐disclosive GLM framework, which includes the Poisson model, and the proportional Cox model without sharing the event times. The current paper aims to fill this gap by providing a tutorial on how time‐to‐event analyses can be performed through PEM using DataSHIELD's existing GLM procedures. For this purpose, it is supposed that the data stored at the different centers are completely and optimally harmonized, which is required before applying federated analysis approaches [22]. The article is outlined as follows: In Section 2, we describe how a Cox model can be approximated by a piecewise exponential model (included in GLM procedures) and why a Cox model presents specific challenges for federated analyses. In Section 3, we explain how PEM is implemented in DataSHIELD illustrated by an example using data available on a demo server. A tutorial with detailed worked examples is provided as Supporting Information. The R code used in Section 3 and in the tutorial is also available at a GitHub repository: https://github.com/bsvennblad/federated_time_to_event.

## 2 Cox Model Versus Piecewise Exponential Model

### 2.1 Time‐To‐Event Analysis

A time‐to‐event analysis necessitates being performed on cohort data that contain information on individuals who are followed up from inclusion in the study until the time of occurrence of an event of interest (i.e., outcome) or right censoring. Individuals in the cohort may be exposed to a risk (or protective) factor and the time‐to‐event analysis aims to contrast the exposed and the unexposed individuals by modeling the time until the event of interest occurs in order to investigate how the exposure is related to the outcome [23]. In the following subsections, we describe the Cox proportional hazards model and the Piecewise Exponential Model using the notations summarized in Table 1.

**TABLE 1:** Notations in the cox and piecewise exponential models.

Notation	Description
$[t_{i-1},\left.t_{i}\right)$	Disjunct intervals covering the follow‐up
$d_{j}$	Binary event indicator for individual *j* (0 if censored, 1 if event happened)
h(t)	hazard function at time *t*
X	vector of covariates
β	vector of coefficients to be estimated
$t_{i}$	*i*:th ordered event time
$R_{i}$	individuals at risk just before the time *t*_*i*_
$\lambda_{i}$	event rate in time interval $\left[t_{i-1},\left.t_{i}\right]\right.$
$n_{i}$	number of events in time interval $\left[t_{i-1},\left.t_{i}\right]\right.$
$y_{i}$	total person time

### 2.2 Modeling Time‐To‐Event Data With Cox Proportional Hazards Model

A popular approach to analyze right censored time‐to‐event data is to use the semiparametric Cox proportional hazards model, in which the hazard function $h_{i}\left(t\right)$ for an individual i to experience the outcome at time t is given by the following expression: $h_{i}\left(t|X_{i},\beta \right)=h_{0}\left(t\right)\exp\left(\beta^{\prime }X_{i}\right)$, where $h_{0}\left(t\right)$ is the baseline hazard, $X_{i}$ is a vector of exposure and potential confounder(s) values for individual i, and β is the vector of the corresponding coefficients. Assuming no ties, ordering the event times $t_{i}$, and leaving the baseline hazard $h_{0}\left(t\right)$ unestimated, the vector of coefficients β is estimated by maximizing the following partial likelihood:

$$L\left(\beta \right)=\prod_{t_{i}}\frac{\exp\left(\beta^{\prime }X_{i}\right)}{\sum_{k\in R_{i}}\exp\left(\beta^{\prime }X_{k}\right)}$$

where $R_{i}$ is the group of individuals who are still at risk just before time $t_{i}$ and is called the risk set at time $t_{i}$, illustrated in Figure 1.

**FIGURE 1:**
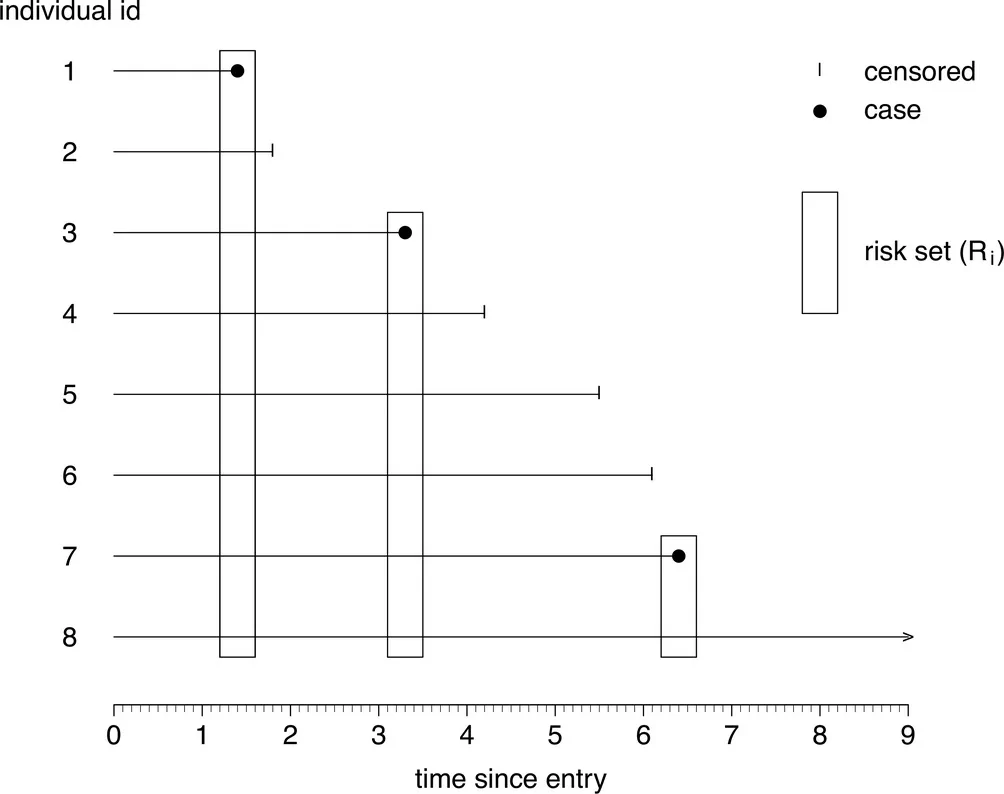
A hypothetical cohort of eight individuals with ordered event and censoring times and the risk set $R_{i}$ at each event time.

The risk set is the central concept in the Cox model and remains the main challenge for implementing federated analyses where no individual‐level data should be shared between different centers. Indeed, given the likelihood's denominator, the number of terms to be added for case i is as large as the number of individuals in the risk set $R_{i}$, which demands sharing (at least some) information on individuals located at different centers.

### 2.3 Modeling Time‐To‐Event Data With Piecewise Exponential Model (Poisson)

The distinct ordered event times are used in the Cox model to index the risk sets. Another option for analyzing right censored time‐to‐event data is to use time as a covariate [24] and model the event rate on discrete time intervals, within the rate is considered constant, using an exponential (i.e., Poisson) model. For the PEM, the constant event rate in time interval *i*, $\lambda_{i}=n_{i}/y_{i}$ is modeled by

$$E\left(\ln(\lambda_{i}|X_{i},\beta \right))=\beta_{0}+\beta^{\prime }X_{i}$$

where $y_{i}$ is the total person time, *n*_*i*_ the number of events, $X_{i}$ is the vector of exposure and potential confounder(s) values for the stratum and time interval i, and β is the vector of the corresponding coefficients. Although time is treated differently in the two approaches, equivalent results would be obtained with the Cox model and the PEM if the time intervals exactly match the time between each event and no ties are present. But, again, this would require access to individual event times, which is not an option for privacy‐preserving federated analyses. Instead, we here approximate the Cox regression by slicing the time scale into (small) time intervals to ensure that the assumption of a constant baseline hazard within each interval can reasonably hold.

The time intervals need to be prespecified, not necessarily equidistant but with intuitively that narrower intervals generally provide a better approximation. The choice of break points for defining the intervals determines the change points of the hazard. Increasing the number of intervals increases the number of event rates to estimate, and too narrow intervals can lead to intervals with no or very few events where $\hat{\lambda_{i}}\approx 0$ resulting in a baseline hazard function highly fluctuating and imprecise estimates. Intervals that are too wide on the other hand, might not capture the underlying changes of the hazard and potentially increase bias. Ideally, the choice of the intervals is based on clinically meaningful cut points (e.g., early vs. late postoperative risk) or prior knowledge about the functional form of the hazard function. Another strategy is to use roughly equal number of events in each interval which can be obtained by using quantiles of event times. It is shown in the vignette of the R‐package survival (https://cran.r‐project.org/web/packages/survival/vignettes/approximate.pdf) how a few straight lines, corresponding to the intervals to be chosen, approximate the non‐parametric cumulative hazard function. In [25] it is recommended to start with a few intervals and then examine the estimated coefficients and standard errors to identify monotone trends or sharp changes in the underlying hazard function. In general, the intervals should be chosen such that each interval contains a sufficient number of events to avoid unstable estimation of the baseline hazard. In practice, this consideration is analogous to the heuristic “events per estimated parameter” guidance commonly used in regression modeling, where recommendations of at least 10–20 events per variable have often been suggested [26, 27]. Very sparse intervals may lead to unstable baseline hazard estimates, convergence issues, or inflated standard errors. Specifying time intervals as a numeric variable instead of a categorical variable would result in a less flexible but smoother baseline hazard with fewer parameters to estimate. Since the interval specification is a crucial step in the PEM, sensitivity analyses with alternative interval specifications are highly recommended.

The equivalence of PEM and Cox assumes no ties. PEM estimates rates within intervals and ties are therefore not an issue. For the Cox model, there are algorithms that handle the presence of ties by modifying the contribution to the partial likelihood in slightly different ways. The methods of Breslow and Efron are two examples that include the covariates from all observations that had an event at the specific event time in the numerator and account for the multiple possible orderings of the tied events in the denominator. The two methods differ in the denominator, where Breslow keeps the risk set equal for all the orders, but Efron changes the risk set according to the order of the tied events. With few ties, the estimates from PEM and Cox should be the same, but in the case of a large number of ties, the estimates can slightly differ depending on the choice of ties handling for the Cox model, even when the follow‐up is divided in intervals based on the unique event times.

The Cox and PEM assume non‐informative right censoring. Splitting the follow‐up into intervals makes the proposed federated PEM approach naturally suitable for interval censored data where the exact event/censoring time is not known, only the interval in which it happens. An additional assumption about the time spent in the individuals' last observed interval needs however to be made as it contributes to the log of the survival time. Left truncation (delayed entry) is handled by excluding the intervals prior to the study start.

In the examples in the Supporting Information we have used time‐invariant covariates. Time‐varying covariates fit naturally into the PEM if the change of value happens at the interval breaks as a covariate can have different values for different intervals in the expanded dataset. Non‐proportionality (time‐varying effect) can be modeled by including an interaction term of the covariate and time.

## 3 Implementation of Piecewise Exponential Model in DataSHIELD

### 3.1 Background of DataSHIELD

DataSHIELD is based on a client–server architecture, where each function is a pair of two functions: a client‐ and a server‐side. The server‐side functions are located behind the firewall of the data servers where the data are stored and are those functions that perform the statistical processes applied to the individual‐level data of each single center. Figure 2 A shows a scenario with data stored on 4 separate servers with R and DataSHIELD server‐side functions implemented. A middle‐ware (e.g., Opal [28] or Armadillo [29]) verifies whether a user is authorized to access a given dataset and determines the set of allowed functions. There are two types of server‐side functions, the assign and the aggregate functions. The assign functions run an analytical command and save the output on the server‐side without returning anything to the client‐side. The aggregate functions perform an analytical command and return the output to the client‐side. The outputs from the aggregate server‐side functions pass to the client‐side if and only if the information that is included is non‐disclosive summary statistics filtered through a number of statistical disclosure controls [30]. The client‐side functions are those that call and co‐ordinate the server‐side functions in order to apply a parallel co‐analysis between data from different centers and combine the non‐disclosive information that is returned from the server‐sides. The entire operation procedure confirms that DataSHIELD allows a full analysis of individual‐level data without those data needing to be moved or becoming visible outside of their original location.

**FIGURE 2:**
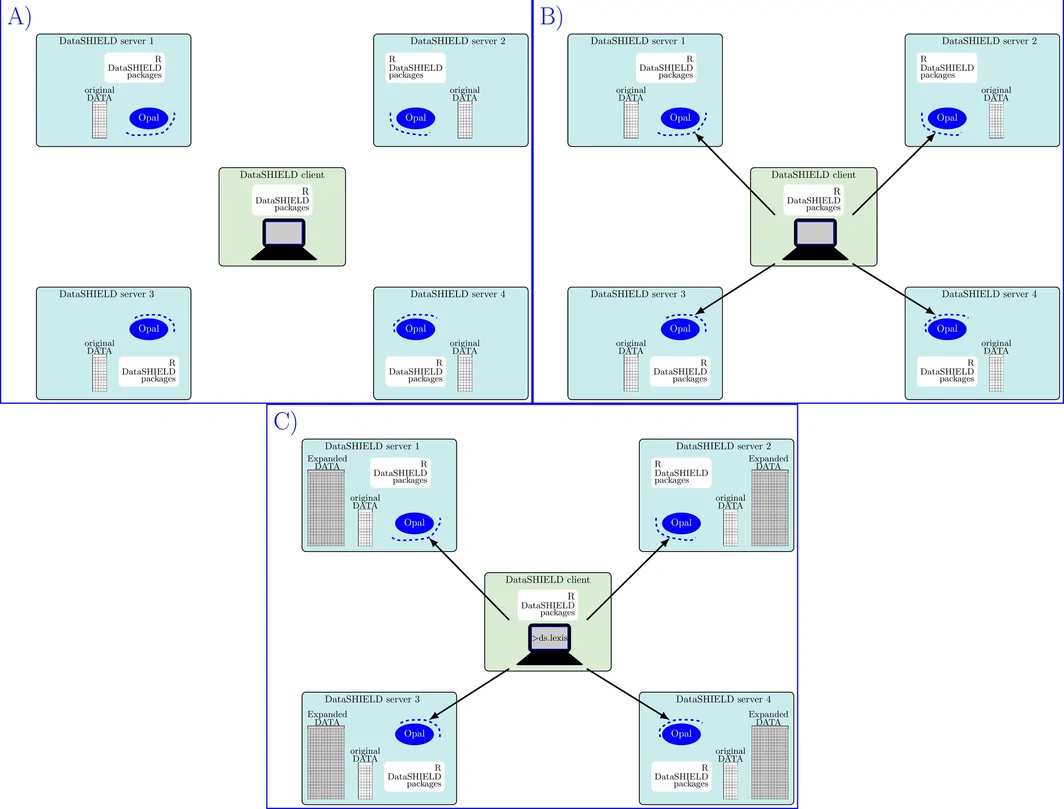
An example of a DataSHIELD infrastructure with 4 centers with harmonized datasets behind fire walls (indicated by dashed lines) where R and server‐side DataSHIELD packages are available (A). The access is controlled, here by Opal, and (B) shows the established connection from the analyst on the client side. In (C) the analyst has used *ds.lexis* to create expanded data sets behind the fire walls in each center.

In addition to the disclosure controls mentioned above and further described below, only DataSHIELD specific commands, not native R, are available to the analyst to increase data protection. Would, however, one of the analyst servers be malicious, thereby violating the honest‐but‐curious trusted setting, some individual data can theoretically be recreated at the client‐side using several functions of particular unusual calculations in specific order iteratively [31]. All DataSHIELD commands performed at a server are recorded permanently in the DataSHIELD log file available only to the data custodian managing the server. By analyzing the log file, it is possible to detect the malicious attack and alert custodians of the disclosure risk. The files can also be used to compare what has been done to the formal governance agreements drawn up when the data were made available for analysis. To further increase privacy protection, pseudonymization is highly recommended, where the additional data needed for identification is kept separately.

### 3.2 Time‐To‐Event Analysis in DataSHIELD

Two functions are used to conduct a time‐to‐event analysis with piecewise exponential regression in DataSHIELD. The first, called *ds.lexis*, performs an assign operation on the server‐side and is responsible for the preparation of the data for the piecewise exponential regression analysis. The probability distribution function belongs to the exponential family, and the PEM is therefore part of the GLM ensemble of models and is among DataSHIELD's available methods. The second function is called *ds.glm*, which has an aggregate server‐side function and is responsible for fitting a generalized linear regression model.

The *ds.lexis* function takes a given data frame that contains the survival data to be analyzed and expands it by converting records at the level of individual subjects (survival time, censoring status, (pseudo‐)IDs and other variables) into multiple records over a series of pre‐defined time intervals (Figure 2B,C). Although the time intervals can be arbitrarily pre‐specified, an objective for the analyst is to choose intervals such that the baseline hazard within each of them is reasonably constant while the baseline hazard can freely vary between the different intervals. For pooled data, as described above, the intervals should be chosen such that each interval contains a sufficient number of events to avoid unstable estimation of the baseline hazard. Fewer and broader intervals, though, might not capture the real changes of the hazard function and potentially increase bias, but increasing the number of intervals increases the number of baseline hazards to be estimated and might overfit the data. Sparse intervals can also be potentially disclosive. The *ds.lexis* function has disclosure checks (see the section Disclosure controls) that block the expansion of the dataset if there is any interval in any center with fewer events than a specified threshold. Even when disclosure thresholds are satisfied, sparse intervals may lead to unstable baseline hazard estimates, convergence issues, or inflated standard errors. Sensitivity analyses using alternative interval specifications, allowed by the disclosure checks, are therefore recommended.

In the expanded data frame multiple rows per individual are created based on the number of intervals that include the follow‐up time of the individual. Each row includes the event status of the individual at a certain time interval, and the survival time indicating the duration of exposure to risk of failure the corresponding individual experienced in that interval before they failed or were censored. The event is represented by a binary variable where a value of 0 indicates that the individual did not experience the event of interest through the interval (passed the upper limit of the interval without failing), and a value of 1 indicates a failure such as death, disease, relapse, and so forth. In addition, the expanded data frame contains all other variables that the analyst would like to carry forward for the survival analysis (e.g., confounders). The individual‐level values of each of those variables are repeated in every row for all time intervals an individual was followed.

Figure 3 shows an example of the conversion of an initial data frame to an expanded data frame in a demonstrative way of what the *ds.lexis* function does. In this example, the initial data frame includes the survival data of the eight hypothetical individuals presented in Figure 1. The expanded data frame in Figure 3 is the transition from the initial data frame by considering the division of the survival time in three intervals that are [0,2), [2,6) and [6,9] (measured in years). It shows that the first individual (ID = 1) dies/fails in the first interval (event is 1 at 1.4 years) while the second individual (ID = 2) is censored in the first interval at 1.8 years (event is 0). The next three individuals (IDs = 3, 4, 5) survive through the first interval and then one individual (ID = 3) dies/fails in the second interval while the other two individuals (IDs = 4, 5) are censored. The exposure‐time variable (survival time) for these three individuals is equal to the length of the first interval (2 years) plus the additional length of time‐to‐event or time‐to‐censoring for each individual in the second interval (1.3, 2.2 and 3.5 years for individuals with IDs 3, 4 and 5 respectively). Adding the survival times together for a particular individual across all time intervals, gives the time when the individual died/failed or censored (e.g., 3.3 years is the time when the individual with ID = 3 failed). Similarly, individuals with IDs = 6, 7 survived during the first two intervals and then either censored (ID = 6 at 6.1 years) or failed (ID = 7 at 6.4 years) after 0.1 and 0.4 years of the third interval, respectively. Finally, the individual with ID = 8 neither failed nor censored in all the three intervals indicating that this individual survived the study's follow‐up which is 9 years in this instance.

**FIGURE 3:**
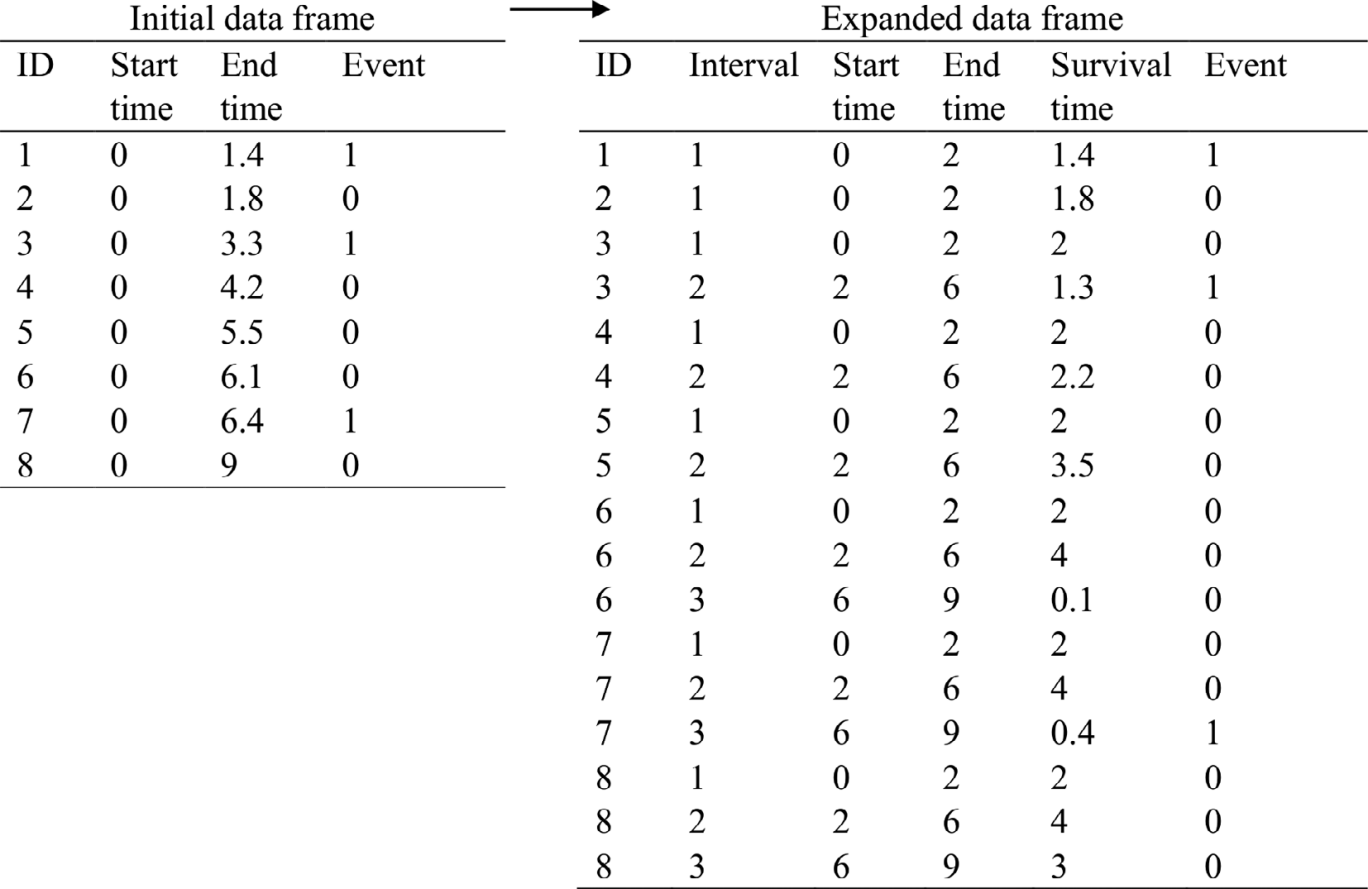
A schematic diagram showing how the time‐to‐event data are expanded from wide to long format based on, pre‐specified by the analyst, time intervals using the *ds.lexis* function. In this specific example the time‐span [0,9] is divided in three intervals that are [0,2), [2,6) and [6,9].

The names of the variables included in the expanded data frame such as the numbered value for each separate time interval, the exposure time and the failure/censoring status in each corresponding interval along with other explanatory covariates are then included in a formula which is used as an input argument of the *ds.glm* DataSHIELD function for the application of the piecewise exponential regression. The event status is used as the outcome variable in the regression model, the value for each separate time period is used as a covariate and the logarithm of the survival time is used as an offset. The offset is effectively a component in the model which has a known coefficient a priori and does not therefore need to be estimated by the regression procedure. For the piecewise exponential regression, the family argument in *ds.glm* is set to “poisson”.

The *ds.glm* function conducts the one‐stage individual participant data analysis in an iterative process where no individual‐level information is transferred or shared between the participating data centers, but only non‐disclosive summary statistics. In the initial step, the client‐side sends a vector of the initial guess for the regression coefficients (by default this is a vector of zeros, see Figure 4A) and requests from each data center to run one iteration of the approximation method used for a maximum likelihood regression analysis. Then, one iteration is performed at each center, and the score vector, *s*, and the information matrix, *I*, are sent back to the client‐side which are both non‐disclosive low‐dimensional summary statistics (Figure 4B). These returned objects from all participating centers are then combined on the client‐side, and an updated vector for the regression coefficients is calculated and sent to the data centers to request the second iteration. The procedure is repeated until it converges on the final solution (Figure 4C).

**FIGURE 4:**
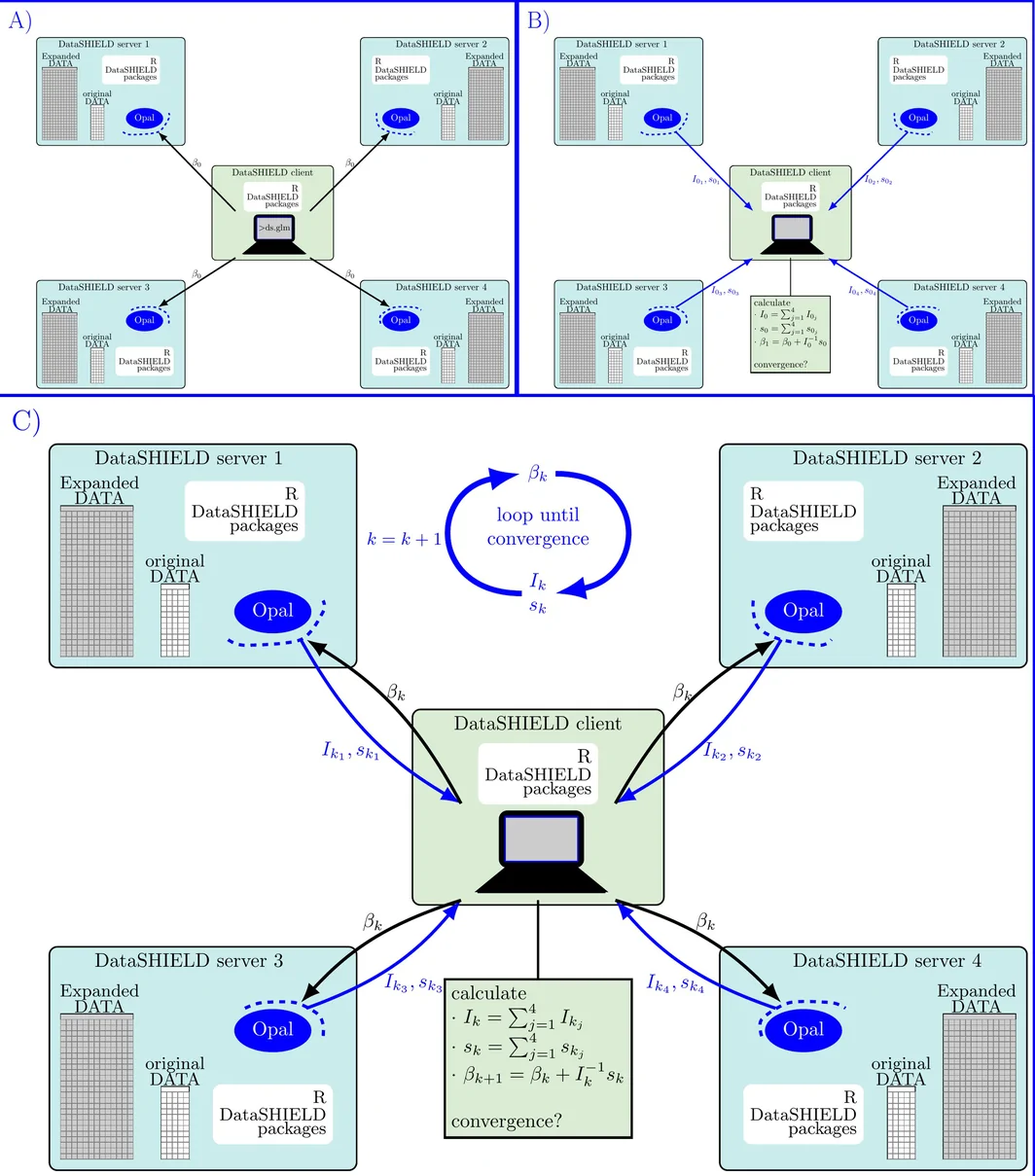
The upper part shows the first iteration conducted by the *ds.glm* function starting by the client‐side initiating the starting value, *β*_0_, of the parameter vector β (A). The server‐side function calculates the center‐specific score vector, S_0j_, and information matrix, I_0j_, and returns them to the client‐side function (B). The client‐side function calculates the total score vector and information matrix as sums of the center‐specific ones, updates the parameter vector *β*, and checks whether convergence is met. If not, the updated vector is sent to the server‐side; center‐specific score vectors and information matrices are calculated and sent to the client‐side function. The procedure is repeated until convergence (C).

### 3.3 Disclosure Controls

Besides the fact that only non‐disclosive summary statistics are returned to the analyst and the raw individual‐level data never become visible to the analyst or shared between the different centers, other disclosure controls are enforced in the server‐side functions to mitigate disclosure risk and allow privacy‐preserving analysis.

One disclosure control enforced by the server‐side function of *ds.lexis* checks the intervals specified by the analyst in the client‐side function to be used for the division of the survival time. If the total sum of the duration across all specified intervals is less than the maximum follow‐up of any individual in any contributing data center, an additional interval is added by *ds.lexis* extending from the end of the last specified interval to the maximum follow‐up time. Also, because the maximum survival times from each center are returned to the client‐side to be combined in one maximum across all centers before the expansion of the data frame (thus to ensure that the end of the final exposure period always includes all of the events in all centers), a positive random number is added to these values to prevent their disclosure.

Even a choice of very poor intervals typically gives relatively similar results to Cox regression analysis. Increasing the number of intervals (and consequently reducing their length) inevitably improves the approximation to the true baseline hazard and to the results of a Cox regression. However, the use of many time intervals, is not only unnecessary but can also become disclosive if the number of failures in one or more of them in a study is small. To prevent such potential disclosure, the number of events in each specified interval should exceed a pre‐specified value set by the study's disclosure control parameter called *nfilter.tab*. This parameter is commonly used in contingency tables to determine the minimum acceptable non‐zero counts in a table's cell. If one or more intervals in a dataset contains fewer failures than the value of this parameter, the expanded data frame is not created, and a corresponding error message is returned to the client‐side. In this case, the analyst can define again the intervals by selecting wider (i.e., fewer) intervals.

Another disclosure control of the server‐side function of *ds.lexis* blocks any attempts to send unpermitted characters to the server‐side as part of the argument for the vector of the intervals widths. It also checks that the length of this vector (consequently the total number of intervals specified) does not exceed a specific threshold defined by the disclosure control parameter, *nfilter.glm* which takes continuous values in (0, 1). If the number of the intervals specified by the analyst is bigger than *nfilter.glm* times the number of rows in the collapsed data frame (i.e., before the expansion to produce the expanded data frame), the expansion is blocked, and an error message is returned to the analyst. This control is important because the argument for the widths of the intervals is set to a numeric vector (completely determined by the user) which might be used to create and define subsets if the used vector has the same length as the primary data vectors to be used in the regression model and potentially to lead to inferential or difference disclosure.

The *nfilter.glm* is also used by the server‐side function of *ds.glm* to block the fitting of a regression model that has more than *nfilter.glm* × N unknown parameters in a dataset with sample size N to prevent the return of potentially disclosive information from over‐saturated models (i.e., models with more terms than the length of the used complete cases dataset). In other words, this is equivalent to the degrees of freedom that the analyst can use in the generalized linear regression model as it specifies the proportion of free parameters that can be used for fitting a model to a certain length of a dataset.

There are default values of the disclosure control parameters which are imported with the installation of the server‐side packages in the servers hosting the data, however, the data providers and custodians are responsible to redefine the specific settings for their dataset usually based on local restrictions and/or risk assessments. By default, *nfilter.tab* is set to 3 which means that *ds.lexis* blocks the expansion of the data frame if any of the specified time intervals at any center include less than 3 events, and *nfilter.glm* is set to 0.33 which means that *ds.glm* blocks the fitting of a regression model with the number of unknown parameters being bigger than 33% of the length of the dataset (i.e., up to 33 parameters in a model allowed to fit a dataset of 100 participants).

### 3.4 Limitations Specific to the Federated Approach

In standard (non‐federated) Cox and PEM analyses might allow for center‐specific baseline hazards, include center as a fixed effect or consider random‐effects extension to allow for heterogeneity across centers. In the federated approach, a factor variable needs to vary within a center to be included in the *ds.glm* model. The call to *ds.glm* will therefore fail with a categorical variable indicating center, but a workaround is to use numerical binary dummy variables (0/1). Center‐specific baseline hazards can be included using interactions between intervals and the dummy variables created to specify center if a numerical function is used for intervals instead of treating intervals as a categorical variable. The number of coefficients to estimate increases rapidly with the increasing number of centers and/or intervals. For a complex model, adding center‐specific baseline hazards might therefore be blocked by the *nfilter.glm* disclosure option. Random‐effects are currently not implemented in DataSHIELD. If heterogeneity is a concern, another option available using DataSHIELD is to fit the *ds.glm* separately in each center and pool the results in a meta‐analysis.

Estimating cause‐specific hazard functions in presence of competing risks is straight forward in PEM by censoring the competing events. The Cumulative Incidence Function estimated through for example, the proportional Fine‐Gray subdistribution model [32] requires weights based on the overall survival curve. In the original paper the Kaplan–Meier estimates were used but that requires the unique event times. An option is to use inverse probability of censoring weighting (IPCW) which is possible using *ds.glm*. There exist algorithms for federated competing risk models using federated learning, for example, ODACoR [33] using multistate models and Fedora [34] based on pseudo values but they both share the individual unique event times. Additionally, the former algorithm needs access to the leading center individual‐level data.

Different residuals (Schoenfeld, Cox‐Snell, martingale etc.) are often used for regression diagnostics in standard Cox. In the federated PEM approach it is possible to create the residuals on the server‐side but the individual‐level values can be disclosive and are therefore not passed to the client‐side. Aggregated information like histograms and residual quantiles can be obtained, center‐specific as well as across centers.

### 3.5 Example Using DataSHIELD

The lung dataset provided in the R‐package survival is split into two equally sized cohorts with the variables described in Table 2. The datasets are available in the project *Poisson_reg* for analyses using DataSHIELD from https://opal‐demo.obiba.org/. The R‐code used in this section is also available at https://github.com/bsvennblad/federated_time_to_event. We have given *dsuser1* and *dsuser2* permission to analyze (but not view) the data in the first and second dataset respectively, mimicking a situation with two different servers. The analyst can connect to server(s) using the credentials of the two users:

**TABLE 2:** Variables in the lung dataset that is available for analyses from https://opal‐demo.obiba.org/.

Variable	Description
Id	Subject Identifier
Entrytime	Entry Time In The Study, Should Be 0
Time	Time Until Death Or Censoring
Status	Event Status (0 = Censored, 1 = Dead)
Age	Age At Baseline
Sex	Gender (0 = Male, 1 = Female)
ph.ecog	ECOG performance score

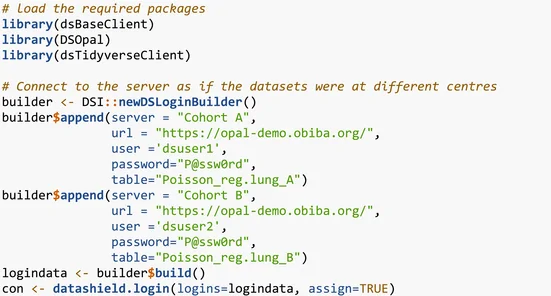

The follow‐up should be split into disjunct intervals where the hazard can be assumed to be constant. Our main purpose in this example is not intended as a valid interpretable analysis but to show how the functions work in practice. We start by trying to split the follow‐up into intervals of length 100.

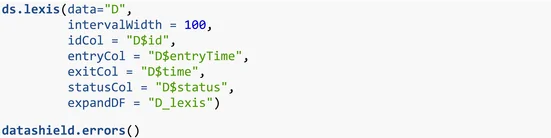

The command failed because the disclosure option *nfilter.tab* blocks *ds.lexis* if there is an interval with too few events. A histogram of the event times might help identify where in the follow‐up this occurs. A subset of the original data containing the observations ending with an event is created on the server‐side and a histogram can then be created.

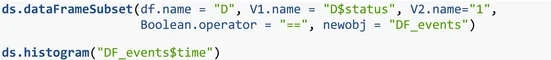

There are too few events when time is large so intervals of length 100 are not allowed in that area (see Figure 5). At the beginning of the follow‐up there are more events, which allows narrower intervals. We specify interval lengths of 30 up to time *t* = 330, and thereafter longer intervals. As described above, *ds.lexis* will add an interval at the end to ensure that the entire follow‐up is covered. The function *ds.dim* gives the number of rows and columns of the *D_lexis* objects created on the server‐side, which is a quick way to verify that they have been created.

**FIGURE 5:**
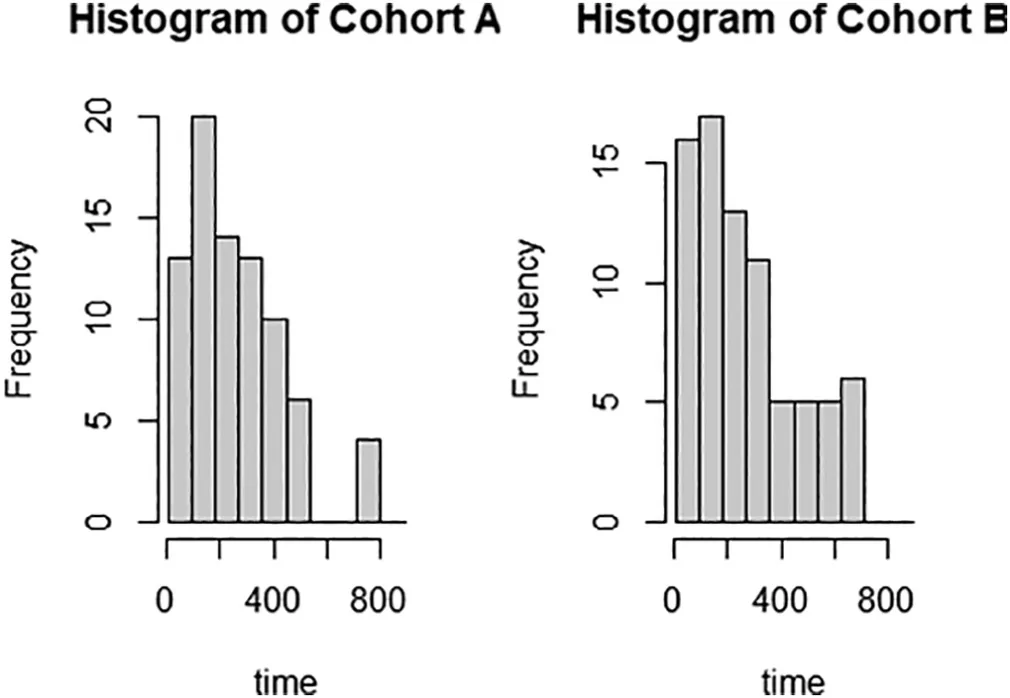
Histogram of event times in cohort A and B respectively as obtained from the DataSHIELD function *ds. histogram*.

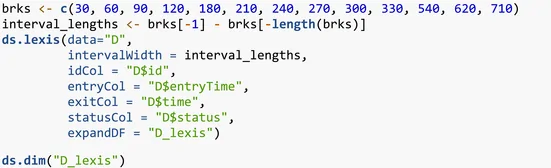

We change the intervals to a factor variable and include it in the dataframe.

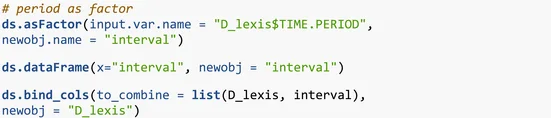

Next, we calculate the log of the time spent in each interval that should be added to the Poisson model as an offset. The model can then be fitted and we save the result on the client‐side in the object *fit.ds*. A summary of the estimated coefficients is shown in Table 3.

**TABLE 3:** Result from the Poisson regression performed on the lung dataset using DataSHIELD.

	Estimate	Std. error	*p*‐value
Interval 1	−7.3597183	0.6700989	0.0000000
Interval 2	−7.6746957	0.6983647	0.0000000
Interval 3	−7.2638728	0.6662582	0.0000000
Interval 4	−7.1789002	0.6279144	0.0000000
Interval 5	−6.6071329	0.6387730	0.0000000
Interval 6	−6.5094364	0.6432237	0.0000000
Interval 7	−6.8798981	0.6783696	0.0000000
Interval 8	−7.1711548	0.7182268	0.0000000
Interval 9	−6.7461767	0.6873368	0.0000000
Interval 10	−6.6282231	0.6115844	0.0000000
Interval 11	−6.3265028	0.6632739	0.0000000
Interval 12	−6.5488110	0.7057675	0.0000000
Interval 13	−6.1659913	0.6557749	0.0000000
Age	0.0162576	0.0091949	0.0770407
Sex 1	−0.5020288	0.1674196	0.0027120

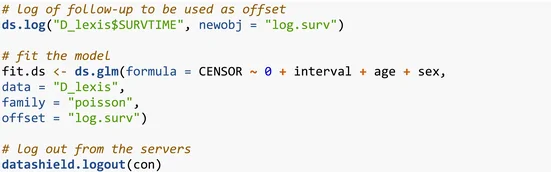

## 4 Discussion

Time‐to‐event analysis is routinely used in medical research and epidemiology for pooled data. For co‐analyzing data located at different centers, one option is to conduct a two‐step meta‐analysis where a separate analysis is done at each center, and the center‐specific results are then pooled and combined into a single estimate. The package *dsSurvival* in DataSHIELD can be used to perform such an analysis for the popular Cox model. For federated individual‐level data, there exist algorithms and implementations of the Cox proportional hazards model with results similar to those that would be obtained if the data were physically pooled. Some of the algorithms construct a surrogate likelihood (ODAC, ODACH), requiring access to the individual‐level data at a dedicated lead center. Other algorithms (WebDISCO, WICOX, DC‐COX) are iterative approaches requiring the individual time points of events to be shared between centers, which can be potentially disclosive.

Poisson regression can be used to approximate the Cox regression without sharing the individual event times and in addition estimate the baseline hazard function, assumed constant within intervals resulting in a step function. The hazard function of the PEM is therefore not as flexible as the hazard function in the Cox model, which is continuous, but the cumulative hazard becomes smoother as the number of intervals increases. The Poisson regression approach can also handle more than one time scale of interest simultaneously.

A crucial step when using PEM for time‐to‐event analyses is to specify the break points that define the consecutive, nonoverlapping intervals of the follow‐up where the baseline hazard can be assumed to be reasonably constant within each interval. There is a trade‐off between finer intervals with few events which can lead to unstable estimates, and broader intervals, which might smooth the hazard function too much. In general, the intervals should be chosen so that there are enough events in each interval to avoid unstable estimation of the baseline hazard. There is no universally accepted minimum number of events per interval, and in practice, the usual recommendation regarding events per estimated parameter is, to our knowledge, the only available guidance. For the federated approach, sparse intervals additionally increase the disclosure risk. A lower threshold for the number of events needed in each interval at each center for the *ds.lexis* function to work in DataSHIELD is given by the disclosure option *nfilter.tab*. To further avoid possibly overfitted models from being disclosive, there is a maximum number of coefficients that can be estimated by the *ds.glm* function which is determined by the number of events and the disclosure option *nfilter.glm*. Specifying a functional form for time using the intervals as a numerical instead of a categorical variable might decrease this issue. Sensitivity analyses using alternative interval specifications are highly recommended.

The computational performance for the federated PEM depends on several factors, including the number of participating centers, the number of time intervals, the number of covariates included in the model, and the number of individuals. The federated GLM procedure as implemented in DataSHIELD relies on iterative communication between the client and the servers, where score vectors and information matrices are exchanged at each iteration. Consequently, communication costs increase with both the number of centers and the number of model parameters, while computational costs increase with the size of the expanded datasets and the complexity of the model.

Because computations are performed in parallel across sites, the runtime of each iteration is primarily determined by the slowest participating server and the associated network latency. Increasing the number of intervals expands the dataset and increases the number of baseline hazard parameters that must be estimated. Similarly, including more covariates in the model increases the dimension of the score vectors and information matrices exchanged during the estimation procedure. The disclosure control parameter *nfilter.glm* also limits computational burden in practice by constraining model complexity.

Although we did not perform formal benchmarking analyses for this tutorial paper, our experience from real‐world data applications suggests that the procedure remains practically feasible for moderately large federated studies because computations are distributed across centers and only low‐dimensional summary statistics are exchanged.

The DataSHIELD framework combines governance procedures, access restrictions, and statistical disclosure controls to reduce disclosure risk while allowing joint analyses of distributed individual‐level data. The approach assumes a trusted infrastructure in which server‐side disclosure controls are correctly enforced and participating parties operate in an honest‐but‐curious setting without malicious collusion. The privacy protection therefore relies primarily on organizational safeguards and controlled release of non‐disclosive summary statistics rather than on formal worst‐case privacy guarantees in the sense of differential privacy. Although it has been shown that mathematically iterative multi‐step algorithms of particular unusual calculations can be created to bypass the privacy and an authorized analyst with malicious intent may be able to recreate some individual‐level information, there have to our knowledge been no cases where malicious attacks on DataSHIELD have been undertaken in this manner. As all the analyses undertaken in any given DataSHIELD session are logged and archived in the data servers, should a serious break occur, it is easy to identify which user was responsible for the break in. An appropriate sanction could then be applied. At the minimum this could involve denying the relevant user future access to DataSHIELD for an appropriate period, or to increase the bite of the sanction one could also deny access to DataSHIELD for any other users from the institution from which that user came. Within a project several authorized analysts can be preferred over a single analyst for increasing collaboration and validating analyses, but the number of authorized analysts should be limited if data leakage is of concern. Further, no sensitive data such as names, tax codes, or banking information should be in the data tables available for the analysts, only the data needed for the analyses. Cryptographic privacy‐preserving approaches such as secure multi‐party computation and homomorphic encryption, as well as formal differential privacy mechanisms, represent complementary directions for strengthening privacy guarantees in federated analyses in general, although typically with additional computational, infrastructural, and implementation complexity.

We have shown that DataSHIELD can be used for federated individual patient time‐to‐event data analyses by estimating a piecewise exponential model through a Poisson model using the functions *ds.lexis* and *ds.glm*. Worked examples using publicly available datasets, mimicking the situation where data are stored in different locations, R and the DataSHIELD functions described in this paper are provided as Supporting Information. The R code used can also be obtained from https://github.com/bsvennblad/federated_time_to_event.

## Funding

The authors have nothing to report.

## Conflicts of Interest

The authors declare no conflicts of interest.

## Supporting information


**Data S1:** Supporting Information.

## Data Availability

Only data sets provided in publicly available R packages are used in the Supporting Information. No data is used in the main paper.
