# Supplementary material for: Federated Time‐to‐Event Data Analysis Using Piecewise Exponential Regression in DataSHIELD
Source: Stat Med. 2026 Oct 18;45(23-24):e70789. doi: 10.1002/sim.70789 (PMC13678026; doi:10.1002/sim.70789)
Supplement: Supplementary file 1 [file SIM-45-0-s001.pdf]

Supplementary materials for 'Federated time-to-event analysis using piecewise exponential regression in DataSHIELD'

A tutorial creating and analysing lexis objects with DataSHIELD

Bodil Svennblad[1], Benedicte Delcoigne[2], Paul Burton[3] and Demetris Avraam[4]

2026-05-20

[1]Department of Surgical Sciences, Medical Epidemiology, Uppsala University, Uppsala, Sweden
[2]Department of Medicine, Clinical Epidemiology Division, Karolinska Institutet, Stockholm, Sweden
[3]Population Health Sciences Institute, Newcastle University, Newcastle upon Tyne, United Kingdom
[4]Section of Epidemiology, Department of Public Health, University of Copenhagen, Copenhagen, Denmark

Contents

Introduction **4**

1 Prerequisites **6**

2 Data **7**

2.1 Load data 7

2.2 Structure of the datasets to be analysed 10

2.3 Split data into two cohorts 11

3 Create virtual servers **13**

3.1 Connect to the servers 15

3.2 Check data 15

4 Time scales of follow-up **18**

4.1 Split follow up along a single time scale 18

4.2 Split follow up along multiple time scales 25

5 Analysing time to event using Poisson regression **33**

5.1 Single time scale 33

5.2 Multiple time scales 38

6 Appendix **42**

Data and virtual servers 42

Analysing the lung data (single time scale) 44

Analysing the nickel dataset (two time scales) 46

Package Info **50**

Introduction

This tutorial is a supplement to the paper *Federated time-to-event analysis using piecewise exponential regression in DataSHIELD* [1] and shows how federated time-to-event data can be analysed using R [2] and DataSHIELD [3]. The main focus is on the *ds.lexis* and *ds.glm* functions, which are part of the base package of DataSHIELD, but we will also use other base package functions and functions from the package *dsTidyverse* [4].

Time-to-event (survival) data can be analysed in several ways including e.g. Cox and piecewise exponential (Poisson) regression. The former divides the follow-up into intervals based on event times while the latter divides the follow-up into intervals of prespecified lengths. Within an interval the hazard is assumed to be constant. Sometimes more than one time scale (e.g age and calendar period) are used in the Poisson model and the follow-up needs to be split according to all the relevant time scales.

With pooled data, the functions *survSplit* and *splitLexis* from the R packages *survival* [5] and *Epi* [6, 7] respectively can be used. The latter works on lexis objects making it possible to split the follow-up into intervals based on several time scales. For horizontally splitted harmonised federated data, where datasets with the same variables are stored in different locations, the DataSHIELD function *ds.lexis* can be used to achieve the splitted follow-up objects on the servers.

It is possible to analyse federated data as if it is pooled without any actual transfer of individual data using DataSHIELD [3]. It requires servers up and running each with a middlee-ware (e.g. Opal [8] or Armadillo [9]) for access control, an R environment, DataSHIELD server-side packages and access to the locally stored data. In addition permission for analysing (but not viewing) the individual-level data must be given for the analyst.

In this tutorial we will use publicly available data and examples, splitting the data into two parts, showing how to make the same analyses with locally stored pooled and with federated data. By using *DSLite* [10] we will mimic the situation where the two datasets are stored in different servers. In a real setting with DataSHIELD we are not allowed to view the individual data points, only to use aggregated summaries, but *DSLite* comes with the advantage of objects actually

being visible. We are therefore in this tutorial able to show what is going on at the server-side.

The different sections describe the data, creates the virtual servers and show examples of how data can be analysed federated. The R code used in the sections is also summarised in the Appendix and is further available at https://github.com/bsvennblad/federated_time_to_event.

Chapter 1

Prerequisites

To follow this tutorial R [2] should be implemented. For convenience we further use RStudio [11] but that is not required.

DataSHIELD functions (and packages) come in pairs - one for the client-side and the other for the server-side. The client-side function requests aggregated summaries from the server-side function, which will do the aggregation and return the result to the client-side.

In this tutorial, where we will use *DSLite* [10] to mimic the federated servers, we will need the client-side package *dsBaseClient* (https://github.com/datashield/dsBaseClient) as well as the corresponding server-side package *dsBase* (https://github.com/datashield/dsBase/). The data management required on the server-side for this tutorial can be done federated using *dsBase* but is easier done using the package *dsTidyverse* [4].

The datasets to be used in this tutorial are the dataset *lung* from the *survival* package [5] and the dataset *nickel* from the *Epi* package [6].

If not yet installed, the required packages can be installed from CRAN and github.

```r
library(devtools)
install_github("datashield/dsBaseClient")
install_github("datashield/dsBase")
install.packages("DSLite")
install.packages("Epi")
install.packages("survival")
install.packages("dsTidyverse")
install.packages("dsTidyverseClient")
```

Chapter 2

Data

Two datasets will be used in this tutorial, the dataset *lung* in the package *survival* [5] and the cohort of nickel smelting workers in South Wales stored in the dataset *nickel* distributed with the R package *Epi* [6].

The datasets are chosen as they are publicly available and used in many examples of survival analyses. We will reanalyse the datasets using Cox regression for illustration, split the follow-up at prespecified time points, analyse the datasets with Poisson regression and compare with the Cox analyses. The datasets will be split into two parts and, by using virtual servers, be analysed as if the data was federated. For the federated approach we will be using *DSLite* [10] which will make it possible to view the objects at different steps of the analyses and compare with the same steps for the locally stored datasets.

Pooled data

- Reanalyse Cox regression
- Split follow-up into prespecified intervals
- Analyse with Poisson regression

Federated data

- Split data into two cohorts
- Create two virtual servers and upload the data
- Split follow-up into prespecified intervals
- Analyse with Poisson regression

2.1 Load data

The dataset *lung* is available as soon as the package *survival* is loaded but the dataset *nickel* from the package *Epi* needs to be separately loaded with the

command *data*.

2.1.1 The dataset lung

```
library(survival)
str(lung)
```

```
## 'data.frame':    228 obs. of  10 variables:
##  $ inst     : num  3 3 3 5 1 12 7 11 1 7 ...
##  $ time     : num  306 455 1010 210 883 ...
##  $ status   : num  2 2 1 2 2 1 2 2 2 2 ...
##  $ age      : num  74 68 56 57 60 74 68 71 53 61 ...
##  $ sex      : num  1 1 1 1 1 1 2 2 1 1 ...
##  $ ph.ecog  : num  1 0 0 1 0 1 2 2 1 2 ...
##  $ ph.karno : num  90 90 90 90 100 50 70 60 70 70 ...
##  $ pat.karno: num  100 90 90 60 90 80 60 80 80 70 ...
##  $ meal.cal : num  1175 1225 NA 1150 NA ...
##  $ wt.loss  : num  NA 15 15 11 0 0 10 1 16 34 ...
```

To avoid confusion with an example where we use age as an additional time scale, we will rename the variable *age* to *age.baseline*

```
names(lung)[names(lung)=="age"] <- "age.baseline"
```

Table 2.1: Description of the variables in the dataset *lung*.

variable	description
inst	institutional identification
time	survival times in days
status	censoring status, 1=censored, 2 = dead
age.baseline	age in years
sex	1=male, 2=female
ph.ecog	ECOG performance score (0=good, 5=dead)
ph.karno	Karnofsky performance score (0=bad, good=100) rated by physician
pat.karno	Karnofsky performance score (0=bad, good=100) rated by patient
meal.cal	Calories consumed at meals
wt.loss	Weight loss in last six months

2.1.2 The dataset nickel

```
library(Epi)
data('nickel')
str(nickel)
```

```
## 'data.frame':    679 obs. of  7 variables:
##  $ id      : num  3 4 6 8 9 10 15 16 17 18 ...
##  $ icd     : num  0 162 163 527 150 163 334 160 420 12 ...
##  $ exposure: num  5 5 10 9 0 2 0 0.5 0 0 ...
##  $ dob     : num  1889 1886 1881 1886 1880 ...
##  $ age1st  : num  17.5 23.2 25.2 24.7 30 ...
##  $ agein   : num  45.2 48.3 53 47.9 54.7 ...
##  $ ageout  : num  93 63.3 54.2 69.7 76.8 ...
```

Table 2.2: Description of the variables in the dataset *nickel*.

variable	description
id	identification number
icd	icd7 codes
exposure	exposure, not used in this example
dob	date of birth
age1st	age at first employment
agein	age at start of follow up
ageout	age at end of follow up

2.2 Structure of the datasets to be analysed

2.2.1 The dataset *lung*

We start by changing the numerical variables *status* and *sex* to binary variables. For the federated analysis we will also need status as a logical variable and the time of entry (which is 0 for all as the time scale is the follow-up time).

```
# select variables time, status, age.baseline, sex and ph.ecog
lung <- lung[, c("time", "status", "age.baseline", "sex",
                 "ph.ecog")]

lung$status <- factor(lung$status, levels=c(1,2),
                      labels=c("alive", "dead"))
lung$sex <- factor(lung$sex, levels=c(1,2),
                   labels = c("male", "female"))
# status as a logical variable
lung$status_logical <- (lung$status=="dead")
# entry time for all individuals
lung$entryTime <- 0
```

2.2.2 The dataset *nickel*

The event of interest is death from lung cancer (ICD code 162, 163). The variable *icd* contains the cause of death information with 0 if still alive at the end of follow-up.

Table 2.3: Number of individuals with different icd codes. The lung cancer deaths will define the event of interest.

	ICD7	n
**alive**		
alive	0	47
**dead**		
lung cancer	162, 163	137
other causes	other than {0, 162, 163}	495
**total**		
total		679

We create a new variable, *lung.cancer* which is the status at the end of follow-up, 1 if death of lung cancer, 0 otherwise (alive or death of any other cause). The variable could be created remotely when data is federated (we will do this

with other variables later on in this tutorial) but for now we prefer to create it prior to splitting the data into two cohorts.

```
nickel$lung.cancer <- ifelse(nickel$icd %in% c(162, 163),
                             1, 0)
table(nickel$lung.cancer)
```

```
##
##   0   1
## 542 137
```

2.3 Split data into two cohorts

The datasets *lung* and *nickel* will both be split in two parts, mimicking the situation where data is collected and kept at different sites. To be able to track individual data through this tutorial we start by creating an id variable for the original data.

```
lung$id.lung <- seq_len(nrow(lung))
nickel$id.nickel <- seq_len(nrow(nickel))
```

2.3.1 The dataset *lung*

We will sample the data into two cohorts with 50% of the data in one cohort and the remaining observations in the other.

```
set.seed(seed=123)  # to make the results reproducible
# sample 50% of the rows
ind <- sample.int(n=nrow(lung),
                  size = floor(nrow(lung)*0.5),
                  replace = F)
# the sampled rows will be cohort A,
lung_A <- lung[ind,]
# the rest of the rows will be cohort B
lung_B <- lung[setdiff(seq_len(nrow(lung)), ind),]
```

2.3.2 The dataset *nickel*

The dataset *nickel* will be split into two cohorts by sampling 40 % of the data to one cohort and the remaining observations to the other.

```
nickel$icd <- as.integer(nickel$icd)

# select 40% of the observations to one cohort
set.seed(seed=1234)  # to make the results reproducible
row.nr <- sample(seq_len(nrow(nickel)),
                 size=ceiling(0.4*nrow(nickel)),
                 replace=F)

# select the corresponding rows as cohort A
cohort_A <- nickel[row.nr,]
# create new cohort specific identification numbers
cohort_A$individ <- seq_len(nrow(cohort_A))

# the rest of the observations to another cohort
cohort_B <- nickel[setdiff(seq_len(nrow(nickel)),
                          row.nr),]
cohort_B$individ <- seq_len(nrow(cohort_B))
```

2.3.3 Summary

We now have the datasets summarised in table 2.4

Table 2.4: Summary of the datasets to be further used in this tutorial.

	n	
dataset	observations	events
lung	228	165
lung_A	114	84
lung_B	114	81
nickel	679	137
cohort_A	272	50
cohort_B	407	87

Chapter 3

Create virtual servers

The datasets with federated data, (cohort_A, cohort_B, lung_A and lung_B) will all be in separate virtual servers. To analyse the data we need the packages for the client-side listed in table 3.1. For the federated analyses to work the corresponding server-side packages need to be installed on the server-side. In this tutorial, where we will use *DSLite* and virtual servers, we need to load also the server-side packages in our R session and upload the packages to the virtual servers when those are created.

Table 3.1: Client- and server-side packages to be used in this tutorial when analysing federated data.

packages			
client side	server side	description	more info
DSLite		A serverless DataSHIELD Interface (DSI) implementation to mimic the behavior of a distant data repository server	https://cran.r-project.org/web/packages/DSLite/vignettes/developing-with-dslite.html
dsBaseClient	dsBase	base functions of DataSHIELD	https://github.com/datashield/ dsBaseClient
dsTidyverseClient	dsTyidyverse	DataSHIELD implementation of selected Tidyverse functions	https://github.com/molgenis/ds-tidyverse

```r
# load packages
library(DSLite)
library(dsBaseClient)
library(dsBase)
# for data management using dataSHIELD
library(dsTidyverse)
library(dsTidyverseClient)
```

```r
# create virtual servers
dslite.server <- newDSLiteServer(tables=list(lungA = lung_A,
                                             lungB = lung_B,
                                             nickelA = cohort_A,
                                             nickelB = cohort_B),
                        config = DSLite::defaultDSConfiguration(
                        include=c("dsBase", "dsTidyverse")))
```

3.1 Connect to the servers

Once the virtual servers are created, we can connect to them in the same way we would do if real servers were used, but the 'url' will be the server object that we have created above.

```r
# connect to the lung data
builder <- DSI::newDSLoginBuilder()
builder$append(server = "server1",
               url = "dslite.server",
               table = "lungA",
               driver = "DSLiteDriver")
builder$append(server = "server2",
               url = "dslite.server",
               table = "lungB",
               driver = "DSLiteDriver")
logindata.dslite.tutorial <- builder$build()

conns <- datashield.login(logindata.dslite.tutorial,
                          assign=T,
                          symbol = "lungData")
```

3.2 Check data

We have assigned the symbol 'lungData' to the tables 'lungA' and 'lungB'. We can e.g. check the dimension of the tables and get the frequency table of the

outcome variable.

```
ds.dim(x='lungData', datasources = conns)
```

```
## $`dimensions of lungData in server1`
## [1] 114   8
##
## $`dimensions of lungData in server2`
## [1] 114   8
##
## $`dimensions of lungData in combined studies`
## [1] 228   8
```

```
ds.table(rvar='lungData$status', datasources = conns)
```

```
## $output.list
## $output.list$TABLE_rvar.by.study_row.props
##                study
## lungData$status   server1   server2
##           alive 0.4761905 0.5238095
##           dead  0.5090909 0.4909091
##           NA          NaN       NaN
##
## $output.list$TABLE_rvar.by.study_col.props
##                study
## lungData$status   server1   server2
##           alive 0.2631579 0.2894737
##           dead  0.7368421 0.7105263
##           NA    0.0000000 0.0000000
##
## $output.list$TABLE_rvar.by.study_counts
##                study
## lungData$status server1 server2
##           alive      30      33
##           dead       84      81
##           NA          0       0
##
## $output.list$TABLES.COMBINED_all.sources_proportions
## lungData$status
## alive  dead    NA
## 0.276 0.724 0.000
##
## $output.list$TABLES.COMBINED_all.sources_counts
## lungData$status
```

```
## alive  dead    NA
##    63   165     0
##
##
## $validity.message
## [1] "Data in all studies were valid"
```

Chapter 4

Time scales of follow-up

For time to event (survival) data we follow the individuals from a starting point until the time of event or loss of follow-up. For the start and end of follow-up we need to specify a time scale, e.g time since start of study, calendar time, age etc. Sometimes we are interested in more than one time scale within the same study.

To be able to analyse the time to event with a Poisson regression the follow-up needs to be split into intervals. If we are interested in a single time scale this can be done for pooled data by e.g. the function *survSplit* in the *survival* package or *splitLexis* on a lexis object in the *Epi* package. The latter function can also handle splits along multiple time scales.

4.1 Split follow up along a single time scale

We will analyse the data according to a single time scale (time since entry) in the dataset *lung*.

4.1.1 Pooled data, using function survSplit

For the locally stored pooled dataset we can split the data with the function *survSplit*. The interval breaks need not to be equidistant but the assumption of constant hazard within an interval should be plausible.

```
lungSplit <- survSplit(Surv(time, status=="dead") ~ id.lung +
                         age.baseline + sex + ph.ecog,
                       cut = c(60, 180, 250, 325, 540, 620, 710),
                       data=lung)
```

```
lung[1:2,]
```

```
##   time status age.baseline  sex ph.ecog status_logical entryTime id.lung
## 1  306   dead           74 male       1           TRUE         0       1
## 2  455   dead           68 male       0           TRUE         0       2
```

```
head(lungSplit, n=9)
```

```
##   id.lung age.baseline  sex ph.ecog tstart time event
## 1       1           74 male       1      0   60     0
## 2       1           74 male       1     60  180     0
## 3       1           74 male       1    180  250     0
## 4       1           74 male       1    250  306     1
## 5       2           68 male       0      0   60     0
## 6       2           68 male       0     60  180     0
## 7       2           68 male       0    180  250     0
## 8       2           68 male       0    250  325     0
## 9       2           68 male       0    325  455     1
```

The variables on the right hand side of the formula in the call to *survSplit* are the variables that are retained when the observations are split along the follow-up time. The left hand side of the formula specifies the time and status variable. The status variable should for an individual be 0 for all intervals but the last where the status variable *event* should be equal the observed value in the original data.

```
table(lung$status)
```

```
##
## alive  dead
##    63   165
```

```
table(lungSplit$event)
```

```
##
##   0   1
## 699 165
```

4.1.2 Pooled data, using a lexis object and function splitLexis

In the *Epi* package a lexis object of the cohort data can be created using the function *Lexis*. Using this function all time scales of interest should be defined.

For the dataset *lung* we will use a single time scale, but we will show later definitions of multiple time scales using the dataset *nickel*.

In the dataset *lung* all individuals are followed from the start (time=0), to the time of event or loss of follow-up. The exit of the time scale is therefore given by the variable *time*, the entry is 0. The exit status is given by the variable *status*.

```
lung.lexis <- Lexis(exit = list(myTs = time),
                    exit.status = status,
                    data=lung[, c("id.lung", "time", "status",
                                  "age.baseline", "sex",
                                  "ph.ecog")])
```

```
## NOTE: entry.status has been set to "alive" for all.
## NOTE: entry is assumed to be 0 on the myTs timescale.
```

```
head(lung.lexis)
```

```
##   lex.id myTs lex.dur lex.Cst lex.Xst id.lung time status age.baseline  sex
##        1    0     306   alive    dead       1  306   dead           74 male
##        2    0     455   alive    dead       2  455   dead           68 male
##        3    0    1010   alive   alive       3 1010  alive           56 male
##        4    0     210   alive    dead       4  210   dead           57 male
##        5    0     883   alive    dead       5  883   dead           60 male
##        6    0    1022   alive   alive       6 1022  alive           74 male
##   ph.ecog
##         1
##         0
##         0
##         1
##         0
##         1
```

The lexis object is the data frame given (here restricted to the variables time, status, age.baseline, sex and ph.ecog) with some additional information summarised in table 4.1.

The actual split along the time scale can then be done using the function *splitLexis*.

```
brks <- c(60, 180, 250, 325, 540, 620, 710)

split.lexis <- splitLexis(lung.lexis,
                          breaks = brks,
```

Table 4.1: The additional variables in the lexis object

variable	description
lex.id	identification number, corresponds to the row number of the original dataset
myTs	start of the time scale we defined
lex.dur	duration of the follow-up
lex.Cst	entry status (current state)
lex.Xst	exit status

```
                              time.scale = "myTs")

split.lexis[split.lexis$id.lung %in% c(159, 207),]
```

```
##   lex.id myTs lex.dur lex.Cst lex.Xst id.lung time status age.baseline    sex
##      159    0      60   alive   alive     159  179   dead           63   male
##      159   60     119   alive    dead     159  179   dead           63   male
##      207    0      60   alive   alive     207  224  alive           55 female
##      207   60     120   alive   alive     207  224  alive           55 female
##      207  180      44   alive   alive     207  224  alive           55 female
##   ph.ecog
##         1
##         1
##         0
##         0
##         0
```

4.1.3 Federated data, using ds.lexis

The function *ds.lexis* for the federated data is inspired by the *splitLexis* function and creates the corresponding data frames on the serve-side. **Note!** The splits are identified in the *splitLexis* function by a vector of breaking points while the interval lengths are given in the the function *ds.lexis*.

We continue using the DSLite servers that we created in chapter 3 and logged into in chapter 3.1. The name of the lexis object that we create on the server-side is given in the argument *expandDF*.

```
intervalLengths <- c(brks[1], brks[-1]-brks[-length(brks)])

ds.lexis(data="lungData",
         intervalWidth = intervalLengths,
         idCol = "lungData$id.lung",
```

```
         entryCol = "lungData$entryTime",
         exitCol = "lungData$time",
         statusCol = "lungData$status_logical",
         expandDF = "lungData.split",
         datasources=conns)
```

In a real setting we cannot view the object created on the sever-side, but with the virtual servers and DSLite we can explore the object using the function *getDSLiteData*.

```
ds_lexis_obj <- getDSLiteData(conns, "lungData.split")
ds_lexis_obj$server1[ds_lexis_obj$server1$id.lung %in% c(159, 207),]
```

```
##       UID.expanded IDSEQ TIME.PERIOD SURVTIME CENSOR idseq.orig id.orig
## 159            159     1           1       60      0          1     159
## 159.1        159.1     1           2      119      1          1     159
## 207            207     2           1       60      0          2     207
## 207.1        207.1     2           2      120      0          2     207
## 207.2        207.2     2           3       44      0          2     207
##       starttime.orig endtime.orig exposure.time.orig cens.orig time status
## 159                0          179                179         1  179   dead
## 159.1              0          179                179         1  179   dead
## 207                0          224                224         0  224  alive
## 207.1              0          224                224         0  224  alive
## 207.2              0          224                224         0  224  alive
##       age.baseline    sex ph.ecog status_logical entryTime id.lung
## 159             63   male       1           TRUE         0     159
## 159.1           63   male       1           TRUE         0     159
## 207             55 female       0          FALSE         0     207
## 207.1           55 female       0          FALSE         0     207
## 207.2           55 female       0          FALSE         0     207
```

In the resulting expanded data frame the original variables are kept together with the additional variables summarised in table 4.2.

Table 4.2: Variables added to the expanded dataset.

variable	description
UID.expanded	unique ID of format x.y there x corresponds to the row x in the original data frame, y relates to the (y+1):th interval
IDSEQ	unique ID for each observation in the original dataset. For all observations in the expanded dataset corresponding to a specific observation in the original dataset all have the same IDSEQ.
TIME.PERIOD	the interval number
SURVTIME	time spent in the interval defined by TIME.PERIOD
CENSOR	status variable. Should be 0 for all intervals except the last one the individual contributes to in which it should reflect the observed outcome

4.1.4 Summary

Table 4.3: Summary of some of the important variables of the splitted datasets along a single time scale using different functions

	locally stored pooled data						federated data		
	survsplit			splitLexis			ds.lexis		
id.lung	tstart	time	event	myTs	lex.dur	lex.Xst	TIME.PERIOD	SURVTIME	CENSOR
159	0	60	0	0	60	alive	1	60	0
159	60	179	1	60	119	dead	2	119	1
207	0	60	0	0	60	alive	1	60	0
207	60	180	0	60	120	alive	2	120	0
207	180	224	0	180	44	alive	3	44	0

4.2 Split follow up along multiple time scales

For a lexis object (package *Epi*) it is possible to split the follow-up along all time scales defined. We will follow the example in [7] and split the dataset *nickel* along the time scales *age* and *tfe* (= time since first employment which can be calculated as *agein-age1st*).

4.2.1 Pooled data

For the locally stored pooled data we can start by creating the lexis object, defining the two time scales.

```
# define the lexis object
nickel.lexis <- Lexis(entry = list("age"=agein, "tfe"=agein-age1st),
                      exit = list("age" = ageout),
                      exit.status = lung.cancer,
                      id = id.nickel,
                      data=nickel)
```

```
## NOTE: entry.status has been set to 0 for all.
```

```
head(nickel.lexis)
```

```
##   lex.id   age   tfe lex.dur lex.Cst lex.Xst id icd exposure     dob age1st
##        1 45.23 27.75   47.75       0       0  3   0        5 1889.02  17.48
##        2 48.27 25.08   15.00       0       1  4 162        5 1885.98  23.19
##        3 52.99 27.75    1.17       0       1  6 163       10 1881.25  25.25
##        4 47.91 23.19   21.77       0       0  8 527        9 1886.34  24.72
##        5 54.75 24.79   22.10       0       0  9 150        0 1879.50  29.96
##        6 44.33 23.04   18.21       0       1 10 163        2 1889.91  21.29
##   agein ageout lung.cancer id.nickel
##   45.23  92.98           0         1
##   48.27  63.27           1         2
##   52.99  54.16           1         3
##   47.91  69.68           0         4
##   54.75  76.84           0         5
##   44.33  62.54           1         6
```

We first split the lexis object along the time scale *age*.

```
age.breaks <- seq(40, 80, 10)
nicS1 <- splitLexis(nickel.lexis, "age", breaks=age.breaks)
nicS1[nicS1$id.nickel==1,]
```

```
##   lex.id   age   tfe lex.dur lex.Cst lex.Xst id icd exposure     dob age1st
##        1 45.23 27.75    4.77       0       0  3   0        5 1889.02  17.48
##        1 50.00 32.52   10.00       0       0  3   0        5 1889.02  17.48
##        1 60.00 42.52   10.00       0       0  3   0        5 1889.02  17.48
##        1 70.00 52.52   10.00       0       0  3   0        5 1889.02  17.48
##        1 80.00 62.52   12.98       0       0  3   0        5 1889.02  17.48
##   agein ageout lung.cancer id.nickel
##   45.23  92.98           0         1
##   45.23  92.98           0         1
##   45.23  92.98           0         1
##   45.23  92.98           0         1
##   45.23  92.98           0         1
```

Note that although we have split the dataset based on the variable *age*, all the variables corresponding to the defined time scales (*age* and *tfe*) are now of the time varying format where the value corresponds to the value at the beginning of the follow-up within the interval. We can now continue with the expanded data frame to split along the time scale *tfe*. Each observation in the dataset above that is covered by the intervals defined from the tfe breaks should be split, and both *tfe* and *age* should be changed accordingly.

```
tfe.brks <- c(20, 30)
nicS2 <- splitLexis(nicS1, "tfe", breaks = tfe.brks)
# periods as categorical variables
nicS2$age.cat <- timeBand(nicS2, "age", type="factor")
nicS2$tfe.cat <- timeBand(nicS2, "tfe", type="factor")

nicS2[nicS2$id.nickel==1,]
```

```
##   lex.id   age   tfe lex.dur lex.Cst lex.Xst id icd exposure     dob age1st
##        1 45.23 27.75    2.25       0       0  3   0        5 1889.02  17.48
##        1 47.48 30.00    2.52       0       0  3   0        5 1889.02  17.48
##        1 50.00 32.52   10.00       0       0  3   0        5 1889.02  17.48
##        1 60.00 42.52   10.00       0       0  3   0        5 1889.02  17.48
##        1 70.00 52.52   10.00       0       0  3   0        5 1889.02  17.48
##        1 80.00 62.52   12.98       0       0  3   0        5 1889.02  17.48
##   agein ageout lung.cancer id.nickel  age.cat  tfe.cat
##   45.23  92.98           0         1  (40,50]  (20,30]
##   45.23  92.98           0         1  (40,50] (30,Inf]
##   45.23  92.98           0         1  (50,60] (30,Inf]
##   45.23  92.98           0         1  (60,70] (30,Inf]
##   45.23  92.98           0         1  (70,80] (30,Inf]
##   45.23  92.98           0         1 (80,Inf] (30,Inf]
```

4.2.2 Federated data

Using the same logic as for the pooled data, by sequentially splitting the dataset along multiple time scale, the federated data can be split along multiple time scales using the function *ds.lexis* - but it requires a little bit more work. For the pooled data we showed above that the variables corresponding to all defined time scales were changed to the value at the start of the interval even though only one scale was splitted. For use of *ds.lexis* we need some additional work to get the values correct for all time scales.

```
# start by disconnecting the lung servers we are connected to
datashield.logout(conns)

# connect to the servers with nickel data
builder <- DSI::newDSLoginBuilder()
builder$append(server = "server1",
               url = "dslite.server",
               table = "nickelA",
               driver = "DSLiteDriver")
builder$append(server = "server2",
               url = "dslite.server",
               table = "nickelB",
               driver = "DSLiteDriver")
logindata.dslite.tutorial <- builder$build()

conns <- datashield.login(logindata.dslite.tutorial,
                          assign=T,
                          symbol = "nickelData")
```

```
# split according to time scale age

age.intervals <- c(age.breaks[1],
               age.breaks[-1]-age.breaks[-length(age.breaks)])

ds.lexis(data="nickelData",
         intervalWidth = age.intervals,
         idCol = "nickelData$id.nickel",
         entryCol = "nickelData$agein",
         exitCol = "nickelData$ageout",
         statusCol = "nickelData$lung.cancer",
         expandDF = "nickelData.age",
         datasources=conns)
```

```
ds_lexis_obj <- getDSLiteData(conns, "nickelData.age")
ds_lexis_obj$server2[ds_lexis_obj$server2$id.nickel==1,]
```

```
##     UID.expanded IDSEQ TIME.PERIOD SURVTIME CENSOR idseq.orig id.orig
## 1              1     1           2   4.7727      0          1       1
## 1.1          1.1     1           3  10.0000      0          1       1
## 1.2          1.2     1           4  10.0000      0          1       1
## 1.3          1.3     1           5  10.0000      0          1       1
## 1.4          1.4     1           6  12.9808      0          1       1
##     starttime.orig endtime.orig exposure.time.orig cens.orig id icd exposure
## 1          45.2273      92.9808            47.7535         0  3   0        5
## 1.1        45.2273      92.9808            47.7535         0  3   0        5
## 1.2        45.2273      92.9808            47.7535         0  3   0        5
## 1.3        45.2273      92.9808            47.7535         0  3   0        5
## 1.4        45.2273      92.9808            47.7535         0  3   0        5
##          dob  age1st   agein  ageout lung.cancer id.nickel individ
## 1   1889.019 17.4808 45.2273 92.9808           0         1       1
## 1.1 1889.019 17.4808 45.2273 92.9808           0         1       1
## 1.2 1889.019 17.4808 45.2273 92.9808           0         1       1
## 1.3 1889.019 17.4808 45.2273 92.9808           0         1       1
## 1.4 1889.019 17.4808 45.2273 92.9808           0         1       1
```

Table 4.4: The variable TIME.PERIOD corresponds to the age intervals defined from the age.breaks.

TIME.PERIOD	age interval
1	-Inf - 40
2	40 - 50
3	50 - 60
4	60 - 70
5	70 - 80
6	80 - Inf

To further split the *nickelData.age* along the time scale *tfe* we need the current value of *tfe* for each individual at the beginning of the follow-up within each interval, that is

$$tfe\_in = \begin{cases} agein - age1st & \text{if first observed interval} \\ \text{age at start of interval} - age1st & \text{otherwise} \end{cases} \tag{4.1}$$

We start by specifying the age of the start of follow up within each interval, which can be done using functions from the *dsTidyverse* package [4].

```
# create a vector of age at period start
ds.case_when(
  tidy_expr = list(
    nickelData.age$TIME.PERIOD==1 ~ 0,
    nickelData.age$TIME.PERIOD==2 ~ 40,
    nickelData.age$TIME.PERIOD==3 ~ 50,
    nickelData.age$TIME.PERIOD==4 ~ 60,
    nickelData.age$TIME.PERIOD==5 ~ 70,
    nickelData.age$TIME.PERIOD==6 ~ 80
  ),
  newobj = "interval_start"
)

# start within period or at the period start
ds.if_else(condition = list(
  nickelData.age$agein > interval_start),
  1,
  0,
newobj = "age_as_start")
ds.dataFrame("age_as_start", newobj = "age_as_start")


# add to the data frame
ds.bind_cols(to_combine=list(nickelData.age, interval_start,
                             age_as_start),
             newobj = "nickelData.age")

#  calculate tfe_in
ds.mutate(df.name="nickelData.age",
          tidy_expr = list(age_interval_start =
                             age_as_start * agein + (1-age_as_start)*interval_start,
                           tfe_in = age_interval_start - age1st),
          newobj = "nickelData.age")
```

```
ds_lexis_obj <- getDSLiteData(conns, "nickelData.age")
ds_lexis_obj$server2[ds_lexis_obj$server2$id.nickel==1,]
```

```
##     UID.expanded IDSEQ TIME.PERIOD SURVTIME CENSOR idseq.orig id.orig
## 1              1     1           2   4.7727      0          1       1
## 1.1          1.1     1           3  10.0000      0          1       1
## 1.2          1.2     1           4  10.0000      0          1       1
## 1.3          1.3     1           5  10.0000      0          1       1
## 1.4          1.4     1           6  12.9808      0          1       1
##     starttime.orig endtime.orig exposure.time.orig cens.orig id icd exposure
## 1          45.2273      92.9808            47.7535         0  3   0        5
```

```
## 1.1        45.2273      92.9808           47.7535         0  3   0        5
## 1.2        45.2273      92.9808           47.7535         0  3   0        5
## 1.3        45.2273      92.9808           47.7535         0  3   0        5
## 1.4        45.2273      92.9808           47.7535         0  3   0        5
##          dob  age1st   agein  ageout lung.cancer id.nickel individ ...22
## 1   1889.019 17.4808 45.2273 92.9808           0         1       1    40
## 1.1 1889.019 17.4808 45.2273 92.9808           0         1       1    50
## 1.2 1889.019 17.4808 45.2273 92.9808           0         1       1    60
## 1.3 1889.019 17.4808 45.2273 92.9808           0         1       1    70
## 1.4 1889.019 17.4808 45.2273 92.9808           0         1       1    80
##     age_as_start age_interval_start  tfe_in
## 1              1            45.2273 27.7465
## 1.1            0            50.0000 32.5192
## 1.2            0            60.0000 42.5192
## 1.3            0            70.0000 52.5192
## 1.4            0            80.0000 62.5192
```

We also need the exit time for each observation which can easily be calculated by the sum of *tfe_in* and *SURVTIME*.

```
ds.mutate(df.name="nickelData.age",
          tidy_expr = list(tfe_out = tfe_in + SURVTIME),
          newobj = "nickelData.age")
```

We choose to rename some of the variables prior to split the dataset along the tfe time scale.

```
ds.rename(df.name = "nickelData.age",
          tidy_expr = list(age.period = TIME.PERIOD,
                           event = CENSOR,
                           age.id = UID.expanded),
          newobj = "nickelData.age")
```

Now we split the dataset along the time scale *tfe*.

```
tfe.intervals <- c(tfe.brks[1],
                   tfe.brks[-1] - tfe.brks[-length(tfe.brks)])

ds.lexis(data="nickelData.age",
         intervalWidth = tfe.intervals,
         idCol = "nickelData.age$age.id",
         entryCol = "nickelData.age$tfe_in",
         exitCol = "nickelData.age$tfe_out",
         statusCol = "nickelData.age$event",
         expandDF = "nickelData.age.tfe",
         datasources=conns)
```

```
ds_lexis_obj <- getDSLiteData(conns, "nickelData.age.tfe")
ds_lexis_obj$server2[ds_lexis_obj$server2$id.nickel==1,]
```

```
##     UID.expanded IDSEQ TIME.PERIOD SURVTIME CENSOR idseq.orig id.orig
## 1              1     1           2   2.2535      0          1       1
## 1.5          1.5     1           3   2.5192      0          1       1
## 1.1          1.1     2           3  10.0000      0          2     1.1
## 1.2          1.2     3           3  10.0000      0          3     1.2
## 1.3          1.3     4           3  10.0000      0          4     1.3
## 1.4          1.4     5           3  12.9808      0          5     1.4
##     starttime.orig endtime.orig exposure.time.orig cens.orig age.id IDSEQ.1
## 1          27.7465      32.5192             4.7727         0      1       1
## 1.5        27.7465      32.5192             4.7727         0      1       1
## 1.1        32.5192      42.5192            10.0000         0    1.1       1
## 1.2        42.5192      52.5192            10.0000         0    1.2       1
## 1.3        52.5192      62.5192            10.0000         0    1.3       1
## 1.4        62.5192      75.5000            12.9808         0    1.4       1
##     age.period SURVTIME.1 event idseq.orig.1 id.orig.1 starttime.orig.1
## 1            2     4.7727     0            1         1          45.2273
## 1.5          2     4.7727     0            1         1          45.2273
## 1.1          3    10.0000     0            1         1          45.2273
## 1.2          4    10.0000     0            1         1          45.2273
## 1.3          5    10.0000     0            1         1          45.2273
## 1.4          6    12.9808     0            1         1          45.2273
##     endtime.orig.1 exposure.time.orig.1 cens.orig.1 id icd exposure      dob
## 1          92.9808              47.7535           0  3   0        5 1889.019
## 1.5        92.9808              47.7535           0  3   0        5 1889.019
## 1.1        92.9808              47.7535           0  3   0        5 1889.019
## 1.2        92.9808              47.7535           0  3   0        5 1889.019
## 1.3        92.9808              47.7535           0  3   0        5 1889.019
## 1.4        92.9808              47.7535           0  3   0        5 1889.019
##      age1st   agein  ageout lung.cancer id.nickel individ ...22 age_as_start
## 1   17.4808 45.2273 92.9808           0         1       1    40            1
## 1.5 17.4808 45.2273 92.9808           0         1       1    40            1
## 1.1 17.4808 45.2273 92.9808           0         1       1    50            0
## 1.2 17.4808 45.2273 92.9808           0         1       1    60            0
## 1.3 17.4808 45.2273 92.9808           0         1       1    70            0
## 1.4 17.4808 45.2273 92.9808           0         1       1    80            0
##     age_interval_start  tfe_in tfe_out
## 1              45.2273 27.7465 32.5192
## 1.5            45.2273 27.7465 32.5192
## 1.1            50.0000 32.5192 42.5192
## 1.2            60.0000 42.5192 52.5192
## 1.3            70.0000 52.5192 62.5192
## 1.4            80.0000 62.5192 75.5000
```

4.2.3 Summary

Table 4.5: A subset of the variables created above for id.nickel=1.

pooled data						federated data			
age	age.cat	tfe	tfe.cat	lex.dur	lex.Xst	age.period	TIME.PERIOD	SURVTIME	CENSOR
45.2273	(40,50]	27.7465	(20,30]	2.2535	0	2	2	2.2535	0
47.4808	(40,50]	30.0000	(30,Inf]	2.5192	0	2	3	2.5192	0
50.0000	(50,60]	32.5192	(30,Inf]	10.0000	0	3	3	10.0000	0
60.0000	(60,70]	42.5192	(30,Inf]	10.0000	0	4	3	10.0000	0
70.0000	(70,80]	52.5192	(30,Inf]	10.0000	0	5	3	10.0000	0
80.0000	(80,Inf]	62.5192	(30,Inf]	12.9808	0	6	3	12.9808	0

Chapter 5

Analysing time to event using Poisson regression

When we have time to event data where the follow-up is split into intervals we can use Poisson regression for the analysis by further assuming that the hazard is constant within the interval, that is a piecewise constant hazard.

5.1 Single time scale

The dataset *lung* is used as illustration of analysing time to event data with a single time scale. For comparison we start by a Cox model. The dataset is used as an example in [12] but there, for the lexis object, the unique event times are used as break points. We will instead use prespecified intervals which will make the result similar but not identical to the example in [12].

```
m0.cox <- coxph(Surv(time, status=="dead")~age.baseline + sex,
                data=lung)
summary(m0.cox)
```

```
## Call:
## coxph(formula = Surv(time, status == "dead") ~ age.baseline +
##     sex, data = lung)
##
##   n= 228, number of events= 165
##
##                   coef exp(coef)  se(coef)      z Pr(>|z|)
## age.baseline  0.017045  1.017191  0.009223  1.848  0.06459 .
## sexfemale    -0.513219  0.598566  0.167458 -3.065  0.00218 **
```

```
## ---
## Signif. codes:  0 '***' 0.001 '**' 0.01 '*' 0.05 '.' 0.1 ' ' 1
##
##                exp(coef) exp(-coef) lower .95 upper .95
## age.baseline      1.0172     0.9831    0.9990    1.0357
## sexfemale         0.5986     1.6707    0.4311    0.8311
##
## Concordance= 0.603  (se = 0.025 )
## Likelihood ratio test= 14.12  on 2 df,   p=9e-04
## Wald test            = 13.47  on 2 df,   p=0.001
## Score (logrank) test = 13.72  on 2 df,   p=0.001
```

5.1.1 Pooled data

```
# define the lexis object and split the follow up
# as done in previous chapters
# i) define the lexis object
lung.lexis <- Lexis(exit = list(myTs = time),
                    exit.status = status,
                    data=lung)
```

```
## NOTE: entry.status has been set to "alive" for all.
## NOTE: entry is assumed to be 0 on the myTs timescale.
```

```
# ii) split follow-up

split.lexis <- splitLexis(lung.lexis,
                          breaks = c(60, 180, 250, 325, 540, 620,
                                     710),
                          time.scale = "myTs")

# period
split.lexis$interval <- timeBand(split.lexis, "myTs",
                                 type = "factor")
```

```
# analyse with Cox
m1.cox <- coxph(Surv(myTs, myTs + lex.dur, lex.Xst=="dead")~
                  age.baseline + sex,
                data=split.lexis)
summary(m1.cox)
```

```
## Call:
```

```
## coxph(formula = Surv(myTs, myTs + lex.dur, lex.Xst == "dead") ~
##     age.baseline + sex, data = split.lexis)
##
##   n= 864, number of events= 165
##
##                   coef exp(coef)  se(coef)      z Pr(>|z|)
## age.baseline  0.017045  1.017191  0.009223  1.848  0.06459 .
## sexfemale    -0.513219  0.598566  0.167458 -3.065  0.00218 **
## ---
## Signif. codes:  0 '***' 0.001 '**' 0.01 '*' 0.05 '.' 0.1 ' ' 1
##
##              exp(coef) exp(-coef) lower .95 upper .95
## age.baseline    1.0172     0.9831    0.9990    1.0357
## sexfemale       0.5986     1.6707    0.4311    0.8311
##
## Concordance= 0.603  (se = 0.026 )
## Likelihood ratio test= 14.12  on 2 df,   p=9e-04
## Wald test            = 13.47  on 2 df,   p=0.001
## Score (logrank) test = 13.72  on 2 df,   p=0.001
```

```
# Poisson regression
m1.p <- glm(lex.Xst=="dead" ~ 0 + interval + age.baseline + sex +
              offset(log(lex.dur)),
            family=poisson(),
            data=split.lexis)
summary(m1.cox)
```

```
## Call:
## coxph(formula = Surv(myTs, myTs + lex.dur, lex.Xst == "dead") ~
##     age.baseline + sex, data = split.lexis)
##
##   n= 864, number of events= 165
##
##                   coef exp(coef)  se(coef)      z Pr(>|z|)
## age.baseline  0.017045  1.017191  0.009223  1.848  0.06459 .
## sexfemale    -0.513219  0.598566  0.167458 -3.065  0.00218 **
## ---
## Signif. codes:  0 '***' 0.001 '**' 0.01 '*' 0.05 '.' 0.1 ' ' 1
##
##              exp(coef) exp(-coef) lower .95 upper .95
## age.baseline    1.0172     0.9831    0.9990    1.0357
## sexfemale       0.5986     1.6707    0.4311    0.8311
##
## Concordance= 0.603  (se = 0.026 )
## Likelihood ratio test= 14.12  on 2 df,   p=9e-04
```

```
## Wald test            = 13.47  on 2 df,   p=0.001
## Score (logrank) test = 13.72  on 2 df,   p=0.001
```

5.1.2 Federated data

We use the virtual servers created in chapter 3 and connected to in chapter 3.1. The expanded dataset *lungData.split* was created in chapter 4.1.3.

TIME.PERIOD is a numerical variable and we start by making it a categorical one and add the new variable to the dataset.

```
# period as factor
ds.asFactor(input.var.name = "lungData.split$TIME.PERIOD",
            newobj.name = "interval")
ds.dataFrame(x="interval", newobj = "interval")
ds.bind_cols(to_combine = list(lungData.split, interval),
             newobj = "lungData.split")
```

For the Poisson regression we need to use the log of time spent in each interval as an offset.

```
# log of follow up to be used as offset
ds.log("lungData.split$SURVTIME",
       newobj = "log.surv")
```

Then we can do the analysis.

```
fit.ds <- ds.glm(formula = CENSOR ~ 0 + interval + age.baseline +
                   sex,
                 data = "lungData.split",
                 family = "poisson",
                 offset = "log.surv")
```

5.1.3 Summary

Table 5.1: Estimated coefficients for the different models.

	pooled			federated
	Cox		Poisson regression	
	m0.cox	m1.cox	m1.p	fit.ds
age.baseline	0.0170453318454113	0.0170453318454112	0.0165085357471151	0.0165085356468645
sexfemale	-0.513218517108384	-0.513218517108385	-0.509190743662436	-0.509190746666418
interval(-Inf,60]			-7.51550887997591	-7.51550887299913
interval(60,180]			-7.05084356161702	-7.0508435554013
interval(180,250]			-6.73001656353046	-6.7300166274397
interval(250,325]			-6.78974034495518	-6.7897403377677
interval(325,540]			-6.61158021696382	-6.61158020991398
interval(540,620]			-6.69401391494569	-6.69401390822718
interval(620,710]			-6.02001995709925	-6.02001995008321
interval(710,Inf]			-6.40748355206129	-6.40748354843968

5.2 Multiple time scales

The dataset *nickel* was split along the time scales *age* and *tfe* and will be analysed following the example in [7].

5.2.1 Pooled data

The lexis object *nicS2* created in chapter 4.2.1 was split along the time scales *age* and *tfe*. We will analyse *nicS2* using Poisson regression and start by defining the time periods as factors.

```
nicS2$age.cat <- timeBand(nicS2, "age", type="factor")
nicS2$tfe.cat <- timeBand(nicS2, "tfe", type="factor")
```

As no deaths had occurred before the age of 40, the authors [7] removed the lowest level of the age factor.

```
fit.pooled <- glm(lex.Xst ~ age.cat + tfe.cat +
                    offset(log(lex.dur)),
                  family=poisson(), data=nicS2,
                  subset=(age >= 40))
```

5.2.2 Federated data

We use the virtual servers created in chapter 3 and connected to in chapter 3.1. The expanded dataset *lungData.split* was created in chapter 4.2.2.

We start by removing the first level of the age categories to follow the example of the pooled data. The function *ds.dataFrameSubset* will do the trick.

```
# subset based on comparing age.period with 1
ds.dataFrameSubset(df.name = "nickelData.age.tfe",
                   V1.name = "nickelData.age.tfe$age.period",
                   V2.name = "1",
                   Boolean.operator = '>',
                   newobj = "nickel.sub")
```

```
# check age.period
ds.table("nickel.sub$age.period")
```

```
## $output.list
## $output.list$TABLE_rvar.by.study_row.props
```

```
##                      study
## nickel.sub$age.period   server1   server2
##                    2  0.3948787 0.6051213
##                    3  0.4072978 0.5927022
##                    4  0.4130781 0.5869219
##                    5  0.4097222 0.5902778
##                    6  0.3793103 0.6206897
##                    NA       NaN       NaN
##
## $output.list$TABLE_rvar.by.study_col.props
##                      study
## nickel.sub$age.period    server1    server2
##                    2  0.26254480 0.27344702
##                    3  0.37007168 0.36601705
##                    4  0.23207885 0.22411693
##                    5  0.10573477 0.10353228
##                    6  0.02956989 0.03288672
##                    NA 0.00000000 0.00000000
##
## $output.list$TABLE_rvar.by.study_counts
##                      study
## nickel.sub$age.period server1 server2
##                    2      293     449
##                    3      413     601
##                    4      259     368
##                    5      118     170
##                    6       33      54
##                    NA       0       0
##
## $output.list$TABLES.COMBINED_all.sources_proportions
## nickel.sub$age.period
##      2      3      4      5      6     NA
## 0.2690 0.3680 0.2270 0.1040 0.0315 0.0000
##
## $output.list$TABLES.COMBINED_all.sources_counts
## nickel.sub$age.period
##    2    3    4    5    6   NA
##  742 1014  627  288   87    0
##
##
## $validity.message
## [1] "Data in all studies were valid"
```

We continue by making *age.period* and *TIME.PERIOD* (tfe intervals) as factors.

```
# age and time period as factors
ds.asFactor(input.var.name = "nickel.sub$age.period",
            newobj.name = "age.cat")
ds.dataFrame(x="age.cat", newobj = "age.cat")

ds.asFactor(input.var.name = "nickel.sub$TIME.PERIOD",
            newobj.name = "tfe.cat")
ds.dataFrame(x="tfe.cat", newobj = "tfe.cat")

ds.bind_cols(to_combine = list(nickel.sub, age.cat, tfe.cat),
             newobj = "nickel.sub")
```

We need the log of the survival times to be used as offset. Thereafter we use *ds.glm* to fit the Poisson model.

```
# log of followup to be used as offset
ds.log("nickel.sub$SURVTIME",
       newobj = "log.surv")

# poisson regression
fit.ds <- ds.glm(formula=CENSOR ~ age.cat + tfe.cat,
                 data="nickel.sub",
                 family="poisson",
                 offset="log.surv")
```

5.2.3 Summary

Table 5.2: Point estimates of the coefficients from the Poisson regression for the dataset *nickel* splitted along time scales *age* and tfe, for pooled and federated data using the functions *glm* and *ds.glm* respectively.

	pooled data	federated data
	glm	ds.glm
(Intercept)	-5.9260762	-5.9260762
age.cat(50,60]	0.8975199	0.8975199
age.cat(60,70]	1.0313589	1.0313589
age.cat(70,80]	0.6304630	0.6304630
age.cat(80,Inf]	0.6443933	0.6443933
tfe.cat(20,30]	0.4273835	0.4273835
tfe.cat(30,Inf]	0.6397107	0.6397107

Chapter 6

Appendix

This appendix includes all the R-code used in this tutorial. The code is also available at https://github.com/bsvennblad/federated_time_to_event.

Data and virtual servers

We use the *lung* and *nickel* datasets from the packages *survival* and *Epi* respectively. The datasets are horisontally split into two parts, mimicking the situation with data collection at different centres. We further create virtual servers and upload the expanded datasets and necessary packages.

```
library(survival)
library(Epi)

library(DSLite)
library(dsBaseClient)
library(dsBase)
# for data management using dataSHIELD
library(dsTidyverse)
library(dsTidyverseClient)

#------------------------------------------------------------
# lung data
# select variables time, status, age, sex and ph.ecog
lung <- lung[, c("time", "status", "age", "sex", "ph.ecog")]

# change to binary variables
lung$status <- factor(lung$status, levels=c(1,2),
                      labels=c("alive", "dead"))
```

```
lung$status_logical <- (lung$status=="dead")
lung$sex <- factor(lung$sex, levels=c(1,2),
                   labels = c("male", "female"))

# rename age variable
names(lung)[names(lung)=="age"] <- "age.baseline"

# create a PID
lung$id.lung <- seq_len(nrow(lung))

# entry time 0 for all individuals
lung$entryTime <- 0
# split data horisontally into two parts
set.seed(seed=123)  # to make the results reproducible
# sample 50% of the rows
ind <- sample.int(n=nrow(lung),
                  size = floor(nrow(lung)*0.5),
                  replace = F)
# the sampled rows will be cohort A,
# create a cohortspecific id
lung_A <- lung[ind,]
lung_A$id <- seq_len(nrow(lung_A))
# the rest of the rows will be cohort B
lung_B <- lung[setdiff(seq_len(nrow(lung)), ind),]
lung_B$id <- seq_len(nrow(lung_B))


#-----------------------------------------------------------
# nickel data
data(nickel)
nickel$lung.cancer <- ifelse(nickel$icd %in% c(162, 163),
                             1, 0)
nickel$id.nickel <- seq_len(nrow(nickel))
# select 40% of the observations to one cohort
set.seed(seed=1234)  # to make the results reproducible
row.nr <- sample(seq_len(nrow(nickel)),
                 size=ceiling(0.4*nrow(nickel)),
                 replace=F)

# select the corresponding rows as cohort A
cohort_A <- nickel[row.nr,]
# create new cohort specific identification numbers
cohort_A$individ <- seq_len(nrow(cohort_A))

# the rest of the observations to another cohort
```

```r
cohort_B <- nickel[setdiff(seq_len(nrow(nickel)),
                           row.nr),]
cohort_B$individ <- seq_len(nrow(cohort_B))

#-----------------------------------------------------
# create virtual servers
dslite.server <- newDSLiteServer(tables=list(lungA = lung_A,
                                             lungB = lung_B,
                                             nickelA = cohort_A,
                                             nickelB = cohort_B),
                        config = DSLite::defaultDSConfiguration(
                        include=c("dsBase", "dsTidyverse")))
```

Analysing the lung data (single time scale)

```r
#############################################################
# pooled data, locally stored
#############################################################
# define the lexis object
lung.lexis <- Lexis(exit = list(myTs = time),
                    exit.status = status,
                    data=lung)
# split follow-up

split.lexis <- splitLexis(lung.lexis,
                          breaks = c(60, 180, 250, 325, 540, 620,
                                     710),
                          time.scale = "myTs")

# period
split.lexis$interval <- timeBand(split.lexis, "myTs",
                                 type = "factor")

# analyse
fit.pooled <- glm(lex.Xst=="dead" ~ 0 + interval + age.baseline +
                    sex + offset(log(lex.dur)),
                  family=poisson(),
    data=split.lexis)

#################################################################
## federated data
#################################################################
```

```
options(datashield.errors.print = TRUE)
# connect to the lung data
builder <- DSI::newDSLoginBuilder()
builder$append(server = "server1",
               url = "dslite.server",
               table = "lungA",
               driver = "DSLiteDriver")
builder$append(server = "server2",
               url = "dslite.server",
               table = "lungB",
               driver = "DSLiteDriver")
logindata.dslite.tutorial <- builder$build()

conns <- datashield.login(logindata.dslite.tutorial,
                          assign=T,
                          symbol = "lungData")

# define interval lengths
brks <- c(60, 180, 250, 325, 540, 620, 710)
intervalLengths <- c(brks[1], brks[-1]-brks[-length(brks)])

# split follow up
ds.lexis(data="lungData",
         intervalWidth = intervalLengths,
         idCol = "lungData$id.lung",
         entryCol = "lungData$entryTime",
         exitCol = "lungData$time",
         statusCol = "lungData$status_logical",
         expandDF = "lungData.split",
         datasources=conns)

# period as factor
ds.asFactor(input.var.name = "lungData.split$TIME.PERIOD",
            newobj.name = "interval")
ds.dataFrame(x="interval", newobj = "interval")
ds.bind_cols(to_combine = list(lungData.split, interval),
             newobj = "lungData.split")

# log of follow up to be used as offset
ds.log("lungData.split$SURVTIME",
       newobj = "log.surv")

fit.ds <- ds.glm(formula = CENSOR ~ 0 + interval + age.baseline +
                   sex,
                 data = "lungData.split",
```

```
                  family = "poisson",
                  offset = "log.surv")


datashield.logout(conns)

###############################################################
# compare point estimates
###############################################################

cbind(fit.pooled$coefficients, fit.ds$coefficients[,"Estimate"])
```

Analysing the nickel dataset (two time scales)

```
#################################################################
# pooled data
#################################################################

# define the lexis object
nickel.lexis <- Lexis(entry = list("age"=agein,
                                   "tfe"=agein-age1st),
                      exit = list("age" = ageout),
                      exit.status = lung.cancer,
                      id = id.nickel,
                      data=nickel)
# split follow-up
age.breaks <- seq(40, 80, 10)
nicS1 <- splitLexis(nickel.lexis, "age", breaks=age.breaks)
tfe.breaks <- c(20, 30)
nicS2 <- splitLexis(nicS1, "tfe", breaks = tfe.breaks)

# periods as categorical variables
nicS2$age.cat <- timeBand(nicS2, "age", type="factor")
nicS2$tfe.cat <- timeBand(nicS2, "tfe", type="factor")

fit.pooled <- glm(lex.Xst ~ age.cat + tfe.cat +
                    offset(log(lex.dur)),
                  family=poisson(), data=nicS2,
                  subset=(age >= 40))

################################################################
# federated data
```

```
################################################################

builder <- DSI::newDSLoginBuilder()
builder$append(server = "server1",
               url = "dslite.server",
               table = "nickelA",
               driver = "DSLiteDriver")
builder$append(server = "server2",
               url = "dslite.server",
               table = "nickelB",
               driver = "DSLiteDriver")
logindata.dslite.tutorial <- builder$build()

conns <- datashield.login(logindata.dslite.tutorial,
                          assign=T,
                          symbol = "nickelData")

# split according to time scale age
age.breaks <- seq(40, 80, 10)
age.intervals <- c(age.breaks[1],
               age.breaks[-1]-age.breaks[-length(age.breaks)])

ds.lexis(data="nickelData",
         intervalWidth = age.intervals,
         idCol = "nickelData$id.nickel",
         entryCol = "nickelData$agein",
         exitCol = "nickelData$ageout",
         statusCol = "nickelData$lung.cancer",
         expandDF = "nickelData.age",
         datasources=conns)

# create a vector of age at period start
ds.case_when(
  tidy_expr = list(
    nickelData.age$TIME.PERIOD==1 ~ 0,
    nickelData.age$TIME.PERIOD==2 ~ 40,
    nickelData.age$TIME.PERIOD==3 ~ 50,
    nickelData.age$TIME.PERIOD==4 ~ 60,
    nickelData.age$TIME.PERIOD==5 ~ 70,
    nickelData.age$TIME.PERIOD==6 ~ 80
  ),
  newobj = "interval_start"
)

# start within period or at the period start
```

```
ds.if_else(condition = list(
  nickelData.age$agein > interval_start),
  1,
  0,
newobj = "age_as_start")
ds.dataFrame("age_as_start", newobj = "age_as_start")


# add to the data frame
ds.bind_cols(to_combine=list(nickelData.age, interval_start,
                             age_as_start),
             newobj = "nickelData.age")

#  calculate tfe_in
ds.mutate(df.name="nickelData.age",
          tidy_expr = list(age_interval_start =
                             age_as_start * agein + (1-age_as_start)*interval_start,
                           tfe_in = age_interval_start - age1st),
          newobj = "nickelData.age")

# calculate tfe_out
ds.mutate(df.name="nickelData.age",
          tidy_expr = list(tfe_out = tfe_in + SURVTIME),
          newobj = "nickelData.age")

ds.rename(df.name = "nickelData.age",
          tidy_expr = list(age.period = TIME.PERIOD,
                           event = CENSOR,
                           age.id = UID.expanded),
          newobj = "nickelData.age")

# split along time scale tfe
tfe.brks <- c(20, 30)
tfe.intervals <- c(tfe.brks[1],
                   tfe.brks[-1] - tfe.brks[-length(tfe.brks)])

ds.lexis(data="nickelData.age",
         intervalWidth = tfe.intervals,
         idCol = "nickelData.age$age.id",
         entryCol = "nickelData.age$tfe_in",
         exitCol = "nickelData.age$tfe_out",
         statusCol = "nickelData.age$event",
         expandDF = "nickelData.age.tfe",
         datasources=conns)
```

```
# subset where age.period = 1 excluded
ds.dataFrameSubset(df.name = "nickelData.age.tfe",
                   V1.name = "nickelData.age.tfe$age.period",
                   V2.name = "1",
                   Boolean.operator = '>',
                   newobj = "nickel.sub")

# age and time period as factors
ds.asFactor(input.var.name = "nickel.sub$age.period",
            newobj.name = "age.cat")
ds.dataFrame(x="age.cat", newobj = "age.cat")

ds.asFactor(input.var.name = "nickel.sub$TIME.PERIOD",
            newobj.name = "tfe.cat")
ds.dataFrame(x="tfe.cat", newobj = "tfe.cat")

ds.bind_cols(to_combine = list(nickel.sub, age.cat, tfe.cat),
             newobj = "nickel.sub")

# log of followup to be used as offset
ds.log("nickel.sub$SURVTIME",
       newobj = "log.surv")

fit.ds <- ds.glm(formula=CENSOR ~ age.cat + tfe.cat,
                 data="nickel.sub",
                 family="poisson",
                 offset="log.surv")

datashield.logout(conns)

###############################################################
# compare point estimates
###############################################################

cbind(fit.pooled$coefficients, fit.ds$coefficients[,"Estimate"])
```

Package Info

In this tutorial we used R version 4.4.3 (2025-02-28 ucrt). In addition we used the packages listed below.

	package	loadedversion
dsBase	dsBase	6.3.5
dsBaseClient	dsBaseClient	6.3.5
DSLite	DSLite	1.4.1
dsTidyverse	dsTidyverse	1.2.1
dsTidyverseClient	dsTidyverseClient	1.1.0
Epi	Epi	2.65
survival	survival	3.8-6

Bibliography

[1] Bodil Svennblad, Benedicte Delcoigne, Paul Burton, and Demetris Avraam. *Federated time-to event analysis using piecewise exponential regression in DataSHIELD*. 2025.

[2] R Core Team. *R: A Language and Environment for Statistical Computing*. R Foundation for Statistical Computing. Vienna, Austria, 2024. URL: https://www.R-project.org/.

[3] Amadou Gaye, Yannick Marcon, Julia Isaeva, Philippe LaFlamme, et al. "DataSHIELD: taking the analysis to the data, not the data to the analysis". In: *International Journal of Epidemiology* (2014), pp. 1929–1944. DOI: 10.1093/ije/dyu188.

[4] Tim Cadman, Mariska Slofstra, Stuart Wheater, and Demetris Avraam. *dsTidyverse: 'DataSHIELD' 'Tidyverse' Serverside Package*. R package version 1.0.4. 2025. URL: https://cran.r-project.org/package=dsTidyverse.

[5] Terry M Therneau. *A Package for Survival Analysis in R*. R package version 3.8-3. 2024. URL: https://CRAN.R-project.org/package=survival.

[6] Bendix Carstensen, Martyn Plummer, Esa Laara, and Michael Hills. *Epi: A Package for Statistical Analysis in Epidemiology*. R package version 2.60. 2025. URL: https://CRAN.R-project.org/package=Epi.

[7] Martyn Plummer and Bendix Carstensen. "Lexis: An R Class for Epidemiological Studies with Long-Term Follow-Up". In: *Journal of Statistical Software* 38(5) (2011). DOI: 10.18637/jss.v038.i05.

[8] Dany Doiron, Yannick Marcon, Isabel Fortier, Paul Burton, and Vincent Ferretti. "Software application profile: Opal and mica: Open-source software solutions for epidemiological data management, harmonization and dissemination". In: *International Journal of Epidemiology* 46 (5 2017), pp. 1372–1378. URL: https://doi.org/10.1093/ije/dyx180.

[9] Tim Cadman, Mariska K Slofstra, Marije A van der Geest, Demetris Avraam, et al. "MOLGENIS Armadillo: a lightweight server for federated analysis using DataSHIELD". In: *Bioinformatics* 41 (1 2025). URL: https://doi.org/10.1093/bioinformatics/btae726.

[10] Yannick Marcon. *DSLite: 'DataSHIELD' Implementation on Local Datasets*. R package version 1.4.0. 2022. URL: https://CRAN.R-project.org/package=DSLite.

[11] Posit team. *RStudio: Integrated Development Environment for R*. Posit Software, PBC. Boston, MA, 2024. URL: http://www.posit.co/.

[12] Bendix Carstensen. *Who needs the Cox model anyway?* 2023. URL: https://bendixcarstensen.com/WntCma.pdf.
